# scCAN: single-cell clustering using autoencoder and network fusion

**DOI:** 10.1038/s41598-022-14218-6

**Published:** 2022-06-17

**Authors:** Bang Tran, Duc Tran, Hung Nguyen, Seungil Ro, Tin Nguyen

**Affiliations:** 1 Department of Computer Science and Engineering, University of Nevada, Reno, NV 89557 USA; 2 Department of Physiology and Cell Biology, University of Nevada School of Medicine, Reno, NV 89557 USA

**Keywords:** Computational biology and bioinformatics, Data mining, Data processing, Genome informatics, Software

## Abstract

Unsupervised clustering of single-cell RNA sequencing data (scRNA-seq) is important because it allows us to identify putative cell types. However, the large number of cells (up to millions), the high-dimensionality of the data (tens of thousands of genes), and the high dropout rates all present substantial challenges in single-cell analysis. Here we introduce a new method, named single-cell Clustering using Autoencoder and Network fusion (scCAN), that can overcome these challenges to accurately segregate different cell types in large and sparse scRNA-seq data. In an extensive analysis using 28 real scRNA-seq datasets (more than three million cells) and 243 simulated datasets, we validate that scCAN: (1) correctly estimates the number of true cell types, (2) accurately segregates cells of different types, (3) is robust against dropouts, and (4) is fast and memory efficient. We also compare scCAN with CIDR, SEURAT3, Monocle3, SHARP, and SCANPY. scCAN outperforms these state-of-the-art methods in terms of both accuracy and scalability. The scCAN package is available at https://cran.r-project.org/package=scCAN. Data and R scripts are available at http://sccan.tinnguyen-lab.com/

## Introduction

Advances in microfluidics have enabled the isolation of cells, making it possible to profile individual cells using single-cell sequencing technologies^1,2^. This transcriptome profiling of individual cells holds enormous potential for both basic biology and clinical applications, including the identification of new cell types^3,4^, resolution of the cellular dynamics of developmental processes^5^, and identification of gene regulatory mechanisms that vary between cell subtypes^6^.

Unsupervised clustering of scRNA-seq data is one of the most important analyses because it allows us to identify putative cell types. However, the large number of cells (up to millions) and the high-dimensionality of the data (tens of thousands of genes or features) present substantial challenges to computational methods^7^.

One prominent strategy is to reduce the dimensionality of the data before performing cluster analysis. Methods in this category include SC3^8^, CIDR^9^, pcaReduce^10^, SEURAT2^11^, SIMLR^12^, and SHARP^13^. These methods typically apply dimension reduction techniques such as PCA^14^, t-SNE^15^ and UMAP^16^ to obtain a lower-dimensional representation of the data. Deep-learning-based approaches, including scDeepCluster^17^, scAIDE^18^, SCA^19^, AAE-SC^20^, and scGMAI^21^, often use autoencoders to select important features and to project the data onto a low-dimensional latent space. Next, these clustering methods partition the cells using established clustering algorithms (e.g., k-means, spectral clustering, etc.). Since these dimension reduction techniques are sensitive to sequencing platforms^22^ and dropouts^23^, the quality of clustering results also varies accordingly.

Another strategy is to iteratively search for hierarchical structures over both cells and genes. Methods using this strategy include BackSPIN^24^, SAIC^25^, and Panoview^26^). These methods attempt to iteratively divide cells and genes into sub-groups to maximize cell similarity within each cluster. These methods, however, require excessive computational power (due to the iteration), and overestimate the number of cell types.

Many single-cell methods also utilize community detection algorithms such as Louvain^27^ and Leiden^28^. SEURAT3^29^, SCANPY^30^, and Monocle3^31^ embed community detection algorithms in their analysis pipeline. These methods first convert scRNA-seq data into networks in which cells are nodes and the edges represent similarity among them. Next, they partition the network using community detection algorithms that are known to be fast. The quality of clustering results strongly depends on the construction of the similarity network. Further, although community detection algorithms can produce reasonable results, they often overestimate the number of cell communities (cell types).

Lastly, cluster ensemble is another strategy that aims to aggregate results from multiple clustering models. Methods of this class include SAFE^32^, SAME^33^, and Sc-GPE^34^; these methods selectively combine the results obtained from multiple clustering algorithms, including SC3, CIDR, SEURAT, CIDR, SIMLR, SNN-cliq^35^, SSNN-Louvain^36^, and MPGS-Louvain^37^. One of the main drawbacks of clustering ensemble methods is that they do not scale well for large datasets. Moreover, evaluating the quality of clustering results obtained from each individual method is a difficult task because there is no universally agreed standard on what constitutes good quality clusters in the first place^38^.

Here we introduce scCAN, a single-cell clustering approach that consists of three modules: (1) a non-negative kernel autoencoder to filter out uninformative features, (2) a stacked, variational autoencoder to generate multiple low-dimensional representations of single-cell data, and finally (3) a graph-based technique to determine cell groups from multiple representations. In an extensive analysis using 28 scRNA-seq datasets, we demonstrate that scCAN significantly outperforms state-of-the-art methods in separating cells of different types. We further assess the clustering methods with regards to scalability and robustness against dropouts using simulated datasets. Overall, scCAN is the most robust and accurate method and can analyze most datasets in minutes.

## Methods

**Figure 1:**
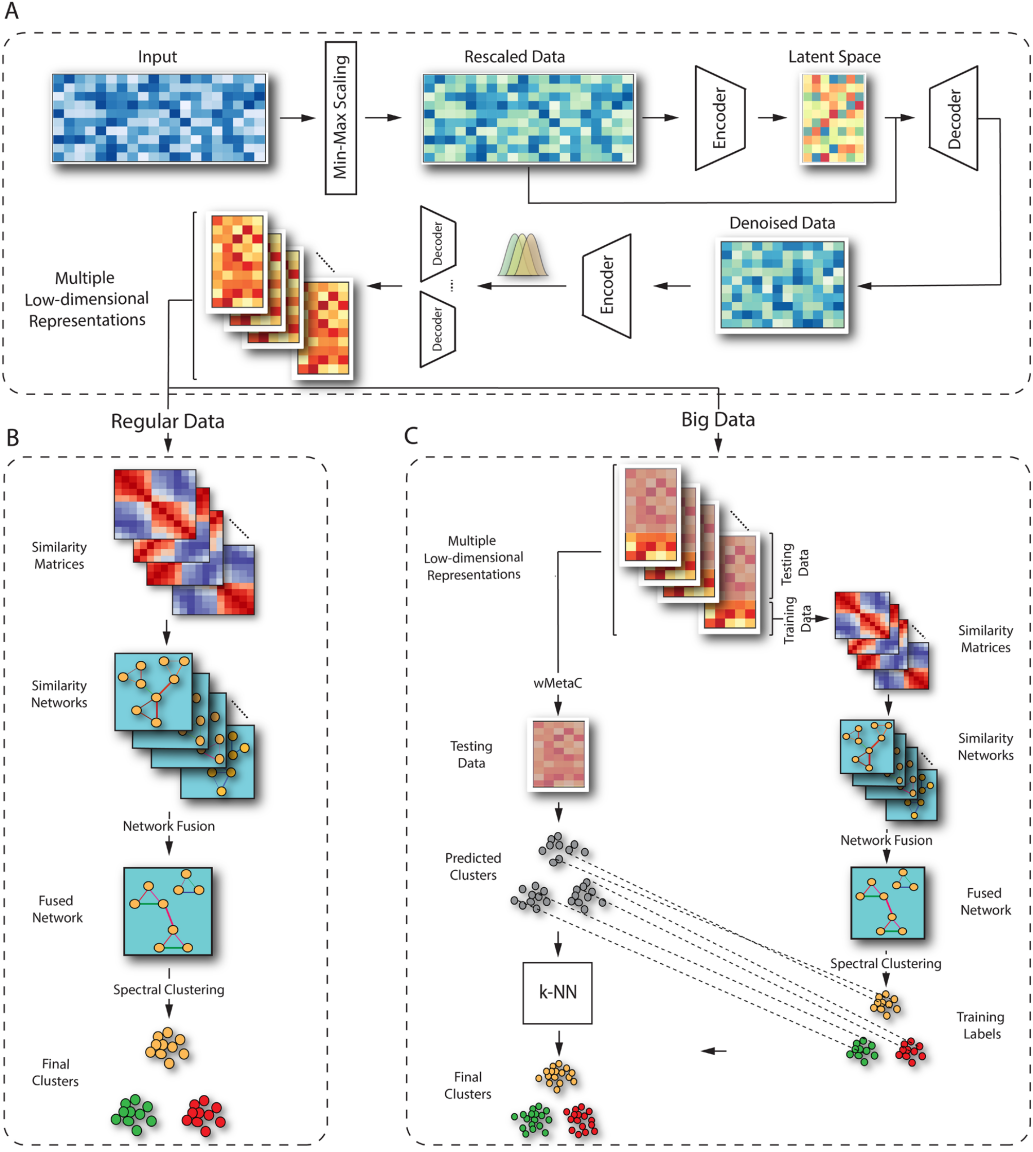
The overall analysis pipeline of scCAN consists of three modules. In the first module (**A**), we perform data normalization, gene filtering, and latent variables generation using two autoencoders. In the second module (**B**), we adopt the network fusion-based clustering method to segregate cell types for small data. The third module (**C**) aims at clustering big data using a combination of the network fusion approach and K nearest neighbors (k-NN) algorithm.

The workflow of scCAN is shown in Fig. 1. This workflow consists of three modules. The first module (Fig. 1A) filters the genes and compresses the input data into a low-dimensional space using two autoencoders. Given the compressed data from module 1, the second module (Fig. 1B) is used to cluster small data, and the third module (Fig. 1C) is used to cluster big data.

### Data compression using autoencoders (Module 1)

Module 1 aims at compressing the original data into a compact representation. This module consists of three main steps: (1) data rescaling, (2) feature selection, and (3) multiple latent variables generation. The first step rescales the data, while the second step removes genes that are not informative. The third step transforms the data obtained from step 2 into a low-dimensional space using a stacked Bayesian autoencoder. The details of each step are presented in the following sections.

#### Min-max scaling

The input of Module 1 is an already-normalized expression matrix in which rows represent cells while columns represent genes. Given the input matrix, we rescale the data to a range of 0 to 1 as follows:

1 $$\begin{aligned} X_{ij} = \dfrac{M_{ij} - min(M_{i.})}{max(M_{i.}) - min(M_{i.})} \end{aligned}$$

where *M* is the input matrix and *X* is the normalized matrix. Note that this min-max scaling is not a scRNA-seq normalization method. This min-max scaling added to our method is used on top of the already normalized data provided by users. Such scaling is frequently used in deep learning models^39–43^ with the common purpose of reducing standard deviation and suppressing the effect of outliers without altering the transcriptome landscape (see Supplementary Section 8 and Figure S11).

#### Feature selection using non-negative-kernel autoencoder

After the rescaling, we further process the data using an 1-layer autoencoder to filter out genes that do not significantly contribute to differentiating cells. Autoencoder is a self-learning neural network that consists of two core components: an encoder and a decoder. The encoder projects the input onto a lower-dimensional space (compressed data) while the decoder tries to reconstruct the original data from the compressed data. Optimizing this process can theoretically result in a compact representation of the original data. By default, we set the dimension of the compressed data (bottleneck layer) to 50. The low number of dimensions ensures that the data obtained from the bottleneck layer is a compact representation of the original input, high-dimensional data.

We also constrain the weights of the encoder to be non-negative, so that each latent variable in the compressed space is a part-based, additive combination of the input. This technique shrinks the coefficients of less important features to zero while maintaining the non-negative coefficients of the significant features. From the weight distribution of the encoder, scCAN only keeps genes that have non-zero coefficients in the part-based representation. In essence, this set of genes can be considered the *optimal set* (sufficient and necessary) to represent the original data. This set is “necessary” because removing any gene from this set would greatly damage the reconstruction ability of the decoder. Concurrently, the set is “sufficient” because adding any other genes would not improve the quality of the compressed data. By default, scCAN selects the top 5000 genes that have non-zero coefficients with the highest coefficient variances.

After this feature selection step, we obtain a new matrix with the same number of cells (rows), but the columns consist of only the optimal set of genes. This matrix serves as the input of another autoencoder to generate multiple low-dimensional representations of the data.

#### Dimensionality reduction using Stacked Variational Autoencoder

After the feature selection step, we obtain a denoised data matrix that consists of important genes. Although a significant number of genes have been removed, there are still thousands of genes. To reduce the computational resources required for clustering, we further reduce the size of the data by conducting an additional step of dimensional reduction using a modified version of Variational Autoencoder (VAE)^44^. We call it Stacked Variational Autoencoder because we generate multiple latent spaces instead of generating only one as in the original VAE.

The VAE has the same structure as a standard autoencoder, which consists of an encoder and a decoder. The encoder ($f_E$) projects the input to a low-dimensional space while the decoder ($f_D$) reconstructs the original input from the compressed data. Given an expression profile of a cell *x*, we have $e=f_E(x)$, where *e* is the low-dimensional representation of *x* in the bottleneck layer. Instead of using *e* directly to reconstruct the data, VAE adds two transformations $f_\mu$ and $f_\sigma$ to generate the parameters $\mu$ and $\sigma$. The new vector *z* is now sampled from the distribution $N(\mu ,\sigma ^2)$. The decoder uses *z* to reconstruct the data: $\bar{x}=f_D(z)$. Adding randomness to *z* will help the VAE model to avoid overfitting without losing the ability of learning a generalized representation of the input.

Here we modify the VAE model to generate multiple compressed spaces with multiple realizations of *z*. The goal is to further diminish overfitting and to increase the robustness of the model. Given a list of latent variables, we use a re-parameterization trick^44^ to obtain multiple realizations of *z* as follows: $z = \mu + \sigma *N(0,1)$. This strategy ensures the VAE model can be back-propagated. In our model, we limit the size of the latent layer to a low number of dimensions (*d* = 15 by default). We keep *d* small to force the neural network to be as compressed as possible.

After finishing the training stage, the input data is processed through the encoder to generate multiple representative latent variables of the original data. As described in the next section, these compressed representations of the data are used for cell segregation (clustering).

### Network fusion and spectral clustering for cell segregation (Module 2)

This section describes the workflow for analyzing datasets with a moderate number of cells ($n<=5000$ by default). When the number of samples is large (over 5000 up to millions of cells), we use a different procedure (see Module 3 in “Big data analysis (Module 3)” section).

The input of Module 2 is multiple low-dimensional representations (matrices) of the input data. We use a network fusion-based approach to cluster scRNA-seq data via multiple steps: (1) building a cell-similarity network for each of the representations, (2) fusing the networks, and (3) clustering using spectral clustering.

For each latent matrix, we construct a cell-similarity network $G = (V,E)$ where each vertex *V* corresponds to a cell and each edge *E* represents a link between two cells. Edges are weighted and stored in a $m \times m$ matrix *W* with $W_{ij}$ represents the weight between cells $x_i$ and $x_j$. To determine the weight for each pair of cells, we first compute the Euclidean distance $\rho _{ij}$ between the cells $x_i$ and $x_j$. Next, we calculate the average value of the distances between the cell $x_i$ and its neighbors $\rho _{i\_} = \frac{\sum _{j=1...k} (\rho (x_i, n_j))}{k}$ . We repeat this step for the cell $x_j$ to obtain $\rho _{j\_}$. We keep the number of neighbors small (*k* = 30 by default) to preserve local cells relationship, but users are free to set their own values. We denote $\varepsilon _{ij} = \frac{\rho _{ij} + \rho _{i\_} + \rho _{j\_}}{3}$ as an average distance among cells $x_i$, $x_j$ and neighbour cells to calculate $W_{ij}=exp\left( -\frac{\rho ^2(x_{i}x_{j})}{\mu \varepsilon _{ij}}\right)$ where $\mu$ is a Gaussian similarity kernel ($\sigma = 0.5$). Finally, we repeat this process for every pair of cells to obtain the similarity matrix W for the current latent matrix to obtain a similarity network. Here, each network is a graph representation of a single latent matrix.

Next, we perform network fusion to aggregate multiple similarity networks obtained from their corresponding latent matrices into a consolidated one. The network fusion approach is adapted from SNF method^45–47^ by first calculating the full and sparse kernel for each vertex *V* in the network *G*. The full kernel is the normalized weight matrix *P* obtained from *G*. The sparse kernel *S* is a matrix that contains local affinity of cells and their neighbors. This step maintains the weights for cells in the same group while suppressing the weights of non-neighbouring cells to zero. That means cell similarities in the same community are more trustworthy than the remote ones. We repeatedly calculate the full and sparse kernel for all *n* similarity networks to get the lists of updated weight matrices and encoded neighbour similarity matrices. Then, those matrices are iteratively fused together to obtain the final fused network *P* as follows:

2 $$\begin{aligned} P^{(v)}= S^{(v)} \times \left( \frac{\sum _{k \ne v} P^{(k)}}{n-1}\right) \times (S^{(v)})^T, \quad v = 1,2,...,n \end{aligned}$$

Given the fused network *P*, we use the eigengap method^48^ to determine the number of clusters. First, we compute adjacency matrix *A* and degree matrix *D* to get Laplacian matrix $L = D-A$. Here, eigen values ($\lambda$) and eigen vectors (*x*) are calculated by $Lx = \lambda x$. Next, eigengap is defined as $eigengap_{i} = \lambda _{i+1} - \lambda _{i}$ where $\lambda _{i}$ is the *i*-th eigenvalue of the matrix *L*. In our method, *i* is user-control hyperparameter that is set from 2 to 15 by default. From the list of eigengap values, we sort them in ascending order and select the two highest eigengap values. Among those two, we select the eigengap that yields a minimum *i* to prevent overestimating the number of clusters. This *i* value is considered as the optimal number of clusters. Given the number of clusters, we use spectral clustering^49^ to partition the cells in the fused network *P*.

### Big data analysis (Module 3)

When the number of cells is large ($n > 5000$), we split the cells into two sets: a training set of 5000 randomly selected cells and a testing set that consists of the remaining cells^50^. We then use the same procedure presented in Module 2 to cluster the *training data*. After this step, we obtain a *training cluster assignment*. We annotate the remaining cells in each latent matrix as *testing data*, and we aim to classify them using the cells labels obtained from the *training data*.

We perform the classification process on *testing data* in only one latent matrix among multiple ones obtained from Module 1. In order to do that, we select the best latent matrix that is a closed representation of other matrices. First, we use k-nearest neighbor adaption of spectral clustering algorithm (k-nn SC) to quickly get the clusters assignments for every latent matrix. Given the list of obtained clusters, we use weighted-based meta-clustering (wMetaC) implemented in SHARP^13^ to determine the final cluster assignment. The wMetaC algorithm is conducted through 5 steps: (1) calculating cell-cell weighted similarity matrix *W*, $w_{ij} = s_{ij}(1-s_{ij})$ where $s_{ij}$ is the chance that cell *i* and *j* are in the same cluster, (2) calculating cell weight, which is the sum of all cell-cell weights related to this cell, (3) generating cluster-cluster similarity matrix $|C|\times |C|$, where *C* is the union of all the clusters obtained in each replicate, (4) performing hierarchical clustering on cluster-cluster similarity matrix, and (5) determining final clustering result by voting scheme. One note of caution is that the final clustering results obtained from this step are only used to determine the best latent matrix. Then, we measure the adjusted Rand index (ARI) value between the final cluster and the cluster obtained from k-nn SC on each input latent. The latent matrix that yields the highest ARI value will be selected for classification.

Given the final latent matrix, we use k-NN algorithm to classify the remaining cells using cluster’s labels obtained from the *training data*. Lastly, we merge the cluster assignments from the *training data* and the *testing data* to get the final clustering result.

Note that the default value of 5000 allows us to have a sufficiently large sample size to properly determine the cell types which in turns will lead to a proper classification of the remaining cells. At the same time, 5000 is a reasonable small number of samples that allows users to perform the analysis efficiently using personal computers. However, this default value might hinder the process of detecting rare cell types in large datasets. To enhance the method’s capability to detect rare cell types, users can either increase the sample size or perform multi-stage clustering. More details are provided in Supplementary Section 9 and Figure S12.

## Results

In this section, we assess the performance of scCAN in the following capabilities: (1) correct estimation of the number of cell types, (2) proper segregation of cells of different types, (3) robustness against dropout events, and (4) scalability against the increasing number of cell types. For this purpose, we analyze 28 real scRNA-seq datasets and simulations in various scenarios. We compare scCAN with five state-of-the-art clustering methods that are widely used for single-cell analysis: CIDR^9^, SEURAT3^29^, Monocle3^31^, SHARP^13^, and SCANPY^30^.

Table 1 shows the number of datasets used in our analysis. Supplementary Table S1 reports more details of the 28 scRNA-seq datasets and Supplementary Table S2 reports the download link. The largest dataset, Brain 1.3M, has 1,300,774 cells. The datasets Guo, Kanton, Brann, and Miller were downloaded from the European Bioinformatics Institute (https://www.ebi.ac.uk/gxa/sc/experiments/). The datasets Slyper, Zilionis, Orozco, and Kozareva were downloaded from Broad Institute Single Cell Portal (https://singlecell.broadinstitute.org/single_cell). The datasets Montoro, Hrvatin, Darrah, Cao were downloaded from NCBI^51^. The Brain 1.3M dataset was downloaded from the 10X Genomics website (https://support.10xgenomics.com/single-cell-gene-expression/datasets/1.3.0/1M_neurons). The remaining 15 datasets were downloaded from Hemberg Group’s website https://hemberg-lab.github.io/scRNA.seq.datasets. We removed samples with ambiguous labels from these datasets. Specifically, we removed cells with the label “zothers” from Chen,“dropped” from Wang, “not applicable” from Segerstolpe, and “Not available” from Guo, Kanton, and Miller. The only processing step we did was to perform a log transformation (base 2) to rescale the data if the range of the data is larger than 100. All datasets except Brain 1.3M have true cell type information and thus can be used to assess the accuracy of the clustering methods. Each of the Cao and Brain 1.3M datasets has more than a million of cells and thus can be used to assess the scalability of the methods.

**Table 1:** Description of the 28 single-cell datasets used in our data analysis.

Name	Accession ID	Tissue	Size	Class	Platform
Brain 1.3M	GSE93421	Mouse brain	1,300,774	NA	10X Genomics
Cao	GSE156793	Human cerebellum	1,092,000	9	10X Genomics
Kozareva	SCP795	Mouse cerebellum	611,034	18	10X Genomics
Darrah	GSE139598	Human blood	162,490	14	Drop-seq
Miller	E-MTAB-8221	Human lung	142,523	11	10X Genomics
Orozco	GSE135133	Human eye	100,055	11	10X Genomics
Hrvatin	GSE102827	Mouse visual cortex	48,266	8	inDrop
Macosko	GSE63473	Mouse retina	44,808	12	Drop-seq
Zilionis	GSE127465	Human lung	34,558	9	inDrop
Brann	E-GEOD-151346	Mouse brain	26,766	46	10X Genomics
Kanton	E-HCAD-5	Human brain	17,542	14	Smart-Seq2
Slyper	SCP345	Human blood	13,316	8	10X Genomics
Chen	GSE87544	Mouse brain	12,089	46	Drop-seq
Baron	GSE84133	Human pancreas	8569	14	inDrop
Guo	E-GEOD-134144	Human testis	7416	7	10X Genomics
Montoro	GSE103354	Human pancreas	7193	7	Smart-Seq2
Lake	phs000833.v3.p1	Human brain	3042	16	Fluidigm C1
Zeisel	GSE60361	Mouse brain	3005	9	STRT-Seq
Romanov	GSE74672	Mouse brain	2881	7	SMARTer
Segerstolpe	E-MTAB-5061	Human pancreas	2209	14	Smart-Seq2
Muraro	GSE85241	Human pancreas	2126	10	CEL-Seq2
Xin	GSE81608	Human pancreas	1600	8	SMARTer
Camp	GSE81252	Human liver	777	7	SMARTer
Usoskin	GSE59739	Mouse brain	622	4	STRT-Seq
Li	GSE81861	Human tissues	561	9	SMARTer
Wang	GSE83139	Human pancreas	457	7	SMARTer
Patel	GSE57872	Human tissues	430	5	Smart-Seq
Pollen	SRP041736	Human tissues	301	11	SMARTer

The table shows the accession ID, tissue, number of cells, true number of cell types, and single-cell platform. All datasets except Brain 1.3M have true cell type information and thus can be used to assess the accuracy of the clustering methods. Each of the Cao and Brain 1.3M datasets has more than a million of cells and thus can be used to assess the scalability of the methods.

We use CIDR^9^, SEURAT3^29^, Monocle3^31^, SHARP^13^, SCANPY^30^, and scCAN to partition each of the 28 real scRNA-seq datasets. CIDR, SEURAT3, Monocle3, and SHARP cannot perform clustering when the dataset has more than 45,000, 30,000, 160,000, and 100,000 cells, respectively. These methods run out of memory in 7, 9, 3, and 5 datasets, respectively (the memory limit is set to 200GB of RAM). Only scCAN and SCANPY can analyze all datasets. Below, we use different metrics to assess the performance of each method.

### Estimating the number of true cell types

We use CIDR^9^, SEURAT3^29^, Monocle3^31^, SHARP^13^, SCANPY^30^, and scCAN to partition each of the 27 real scRNA-seq datasets. To evaluate how well each method estimates the number of cell types, we compare the number of clusters produced by each method against the number of true cell types using the absolute log-modulus^52^: $L(x) = |sign(x)*log10(|x|+1)|$ where *x* is the difference between the number of clusters and the number of cell types. The lower the *L*(*x*) value, the more similar the number of clusters and the true number of cell types. *L*(*x*) equals to zero denotes a perfect estimation.

**Figure 2:**
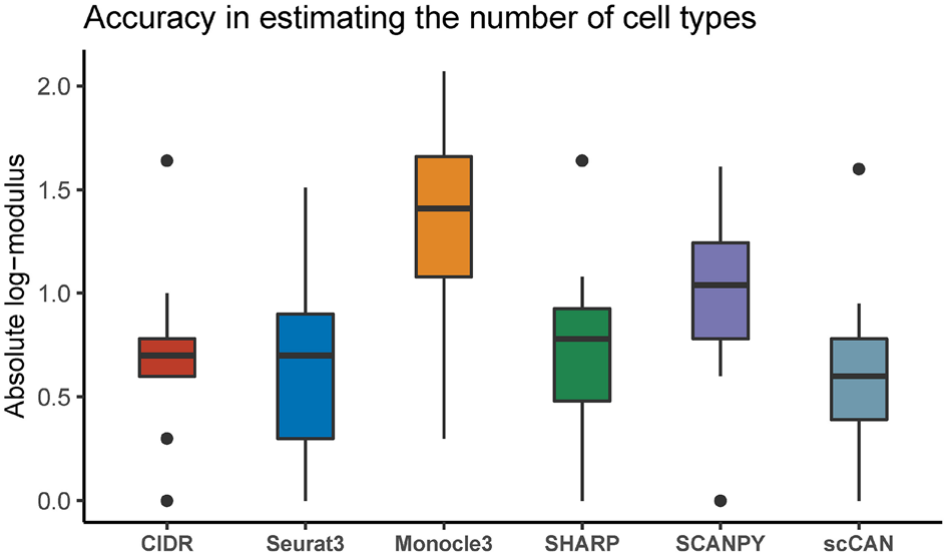
Absolute log-modulus values obtained from CIDR, SEURAT3, Monocle3, SHARP, SCANPY, and scCAN for 27 real scRNA-seq datasets. This metric measures the difference between the number of clusters and the number of true cell types. The average log modulus of scCAN is 0.59 while those of Monocle3, SCANPY, SHARP, SEURAT3, and CIDR are 1.35, 1, 0.72, 0.64, and 0.63, respectively. scCAN significantly outperforms other methods by having the smallest absolute log-modulus values (Wilcoxon *p*-value of $p=8.6\times 10^{-4}$). Note that the dataset Brain 1.3M was excluded from this analysis because it does not have true cell type information.

Figure 2 shows the absolute log-modulus values obtained using the six clustering methods. Each box represents the absolute log-modulus values across 27 scRNA-seq datasets for a method. We observe that Monocle3 and SCANPY frequently overestimate the number of clusters. Both methods have the highest absolute log-modulus values. Overall, scCAN is the best method in estimating the number of true cell types. The average log modulus of scCAN is 0.59 whereas those of Monocle3, SCANPY, SHARP, SEURAT3, and CIDR are 1.35, 1, 0.72, 0.64, and 0.63, respectively. A one-sided Wilcoxon test also confirms that the absolute log-modulus values obtained from scCAN are significantly smaller than other methods with a p-value of $9\times 10^{-4}$. We report the absolute log-modulus values for each method and each dataset in Supplementary Table S3.

### Segregating cells of different types

To assess the accuracy of each clustering method, we also compare the clustering results against the true cell labels. For this purpose, we use three evaluation metrics: adjusted Rand index (ARI)^53^, adjusted mutual information (AMI)^54^, and V-measure^55^. Details of each metric are provided in Supplementary Section 3.

**Figure 3:**
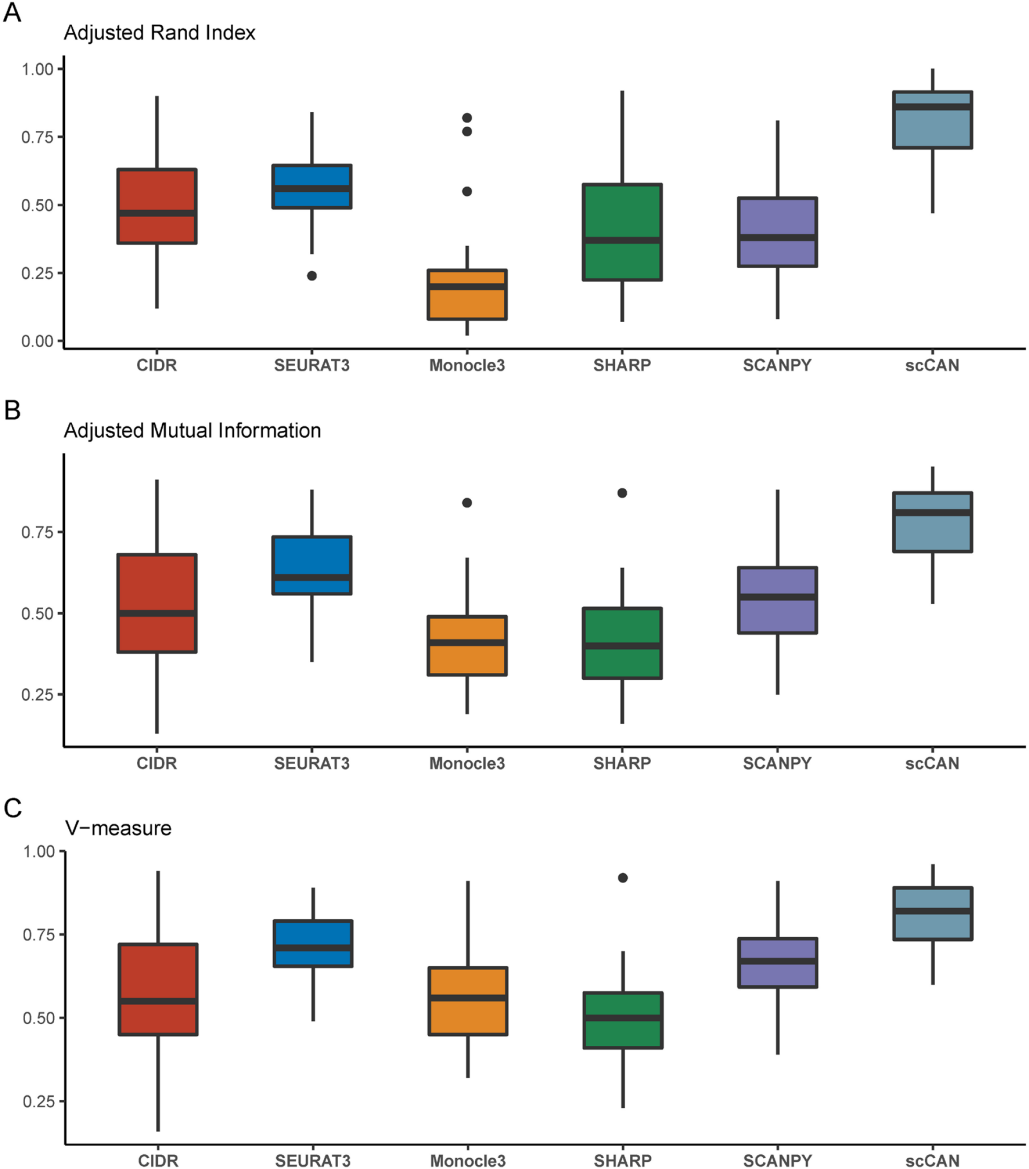
Accuracy assessment of the six clustering methods using adjusted Rand index (ARI), adjusted mutual information (AMI), and V-measure. scCAN consistently and substantially outperforms other methods in every assessment by having the highest ARI, AMI, and V-measure values across 27 real scRNA-seq datasets.

Figure 3A shows the ARI values obtained from the six clustering methods. Each box represents the ARI values across 27 datasets for a method. The results show that scCAN significantly outperforms other state-of-the-art methods by having the highest ARI values ($p=6\times 10^{-12}$ using Wilcoxon test). The average ARI value of scCAN is 0.81 which is substantially higher than those of other methods (0.50, 0.55, 0.23, 0.41, and 0.40 for CIDR, Seurat3, Monocle3, SHARP, and SCANPY, respectively). More importantly, scCAN has the highest ARI values in 24 out of 27 datasets (Supplementary Table S4).

Figure 3B shows the AMI values of each method. The AMI values of scCAN are significantly higher than those of other methods ($p=9 \times 10^{-10}$ using Wilcoxon test). The average AMI value of scCAN is 0.77 while the average AMI values of CIDR, Seurat3, Monocle3, SHARP, and SCANPY are 0.52, 0.64, 0.43, 0.41 and 0.55, respectively. scCAN also has the highest AMI values in 23 out of 27 datasets (Supplementary Table S5).

Figure 3C shows a similar trend using V-measure. The V-measure values of scCAN are significantly higher than those of other methods ($p=2\times 10^{-8}$). The average V-measure value of scCAN is 0.81 while the average AMI values of CIDR, Seurat3, Monocle3, SHARP, and SCANPY are 0.57, 0.72, 0.56, 0.50 and 0.66, respectively. scCAN also has the highest V-measure values in 23 out of 27 datasets (Supplementary Table S6). The visualizations of cell transcriptomic landscape for 27 datasets using original cell types and cluster assignments generated by scCAN are shown in Supplementary Figures S1–S5 and Supplementary Figures S6–S10.

### Robustness against dropouts

One of the prominent challenges in single-cell data analysis is the prevalence of dropouts. To assess how robust each method is against dropouts, we simulate a number of datasets. There are a number of tools that generate simulated data, including Splatter^56^ and SymSim^57^. Though powerful, these tools cannot completely emulate real-world situations. The simulators do not preserve expression levels and gene correlation structure of real genes^58^. Therefore, instead of generating completely new expression values, we simulate different dropout scenarios using the 27 real datasets listed above. For each dataset, we gradually increase the number of dropouts by randomly replacing non-zero expression values with zeros. The dropout rates are set to 50%, 55%, 60%, 65%, 70%, 75%, 80%, 85% and 90%. In summary, we generate 243 simulated datasets (27 real datasets with 9 different dropout rates per dataset).

**Figure 4:**
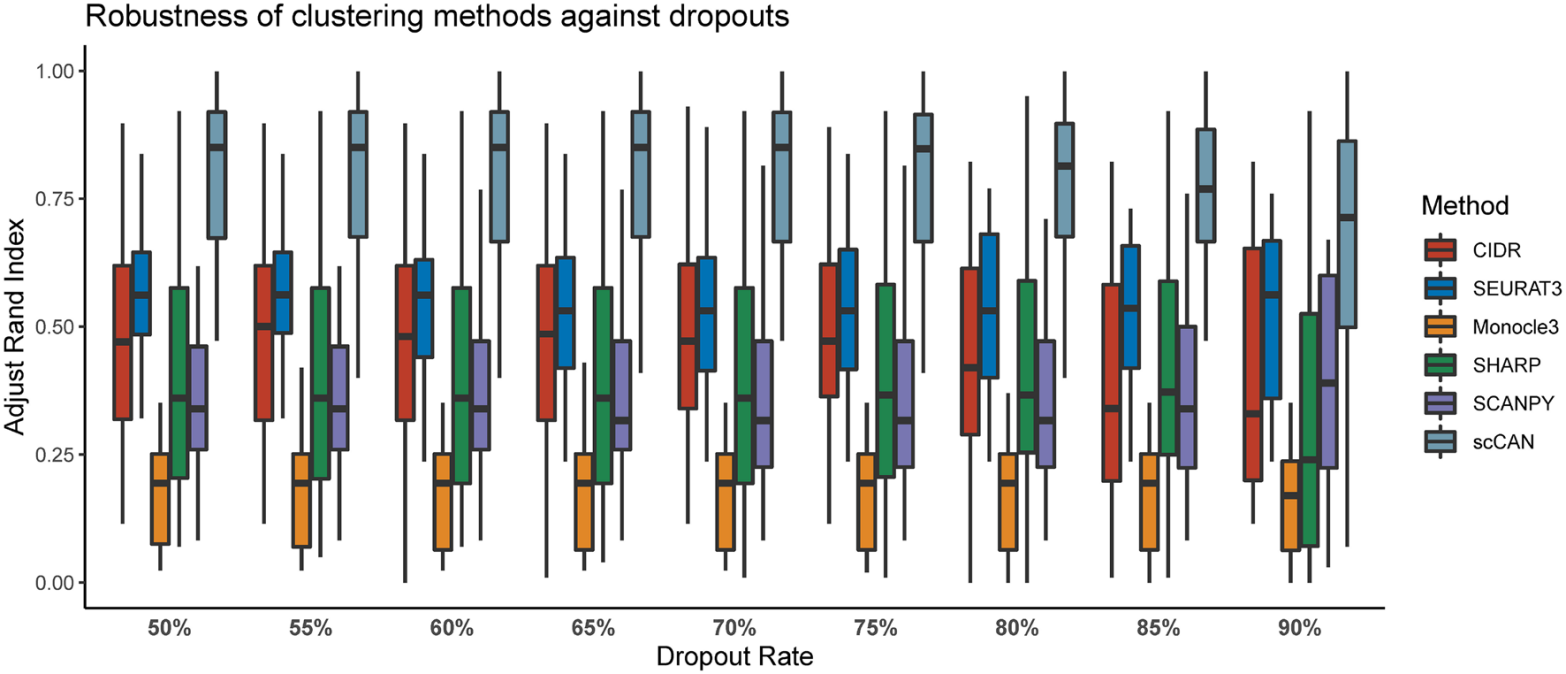
Assessment of CIDR, SEURAT3, Monocle3, SHARP, SCANPY and, scCAN against dropouts. Simulations were obtained by varying the number of zeros in each of 27 real biological datasets from 50% to about 90%, respectively. Each box plot shows the ARI values obtained from each method for a specific dropout portion. Wilcoxon test shows that the ARI values obtained from scCAN are significantly higher than CIDR, SEURAT3, Monocle3, SHARP, SCANPY ($p<2.2 \times 10^{-16}$).

For each dataset, the true cell label of each cell is known and thus can be used *a posteriori* to assess the robustness of each clustering method. We analyze each of the 243 datasets using the six clustering methods and then calculate the ARI values. Figure 4 shows the ARI values for each method across datasets of varying dropout rates. Overall, scCAN consistently outperforms other methods in clustering cell populations regardless of dropout rates. A one-sided Wilcoxon test also confirms that the ARI values obtained from scCAN are significantly higher than those of CIDR, SEURAT3, Monocle3, SHARP, SCANPY ($p<2.2 \times 10^{-16}$).

### Time and space complexity

**Figure 5:**
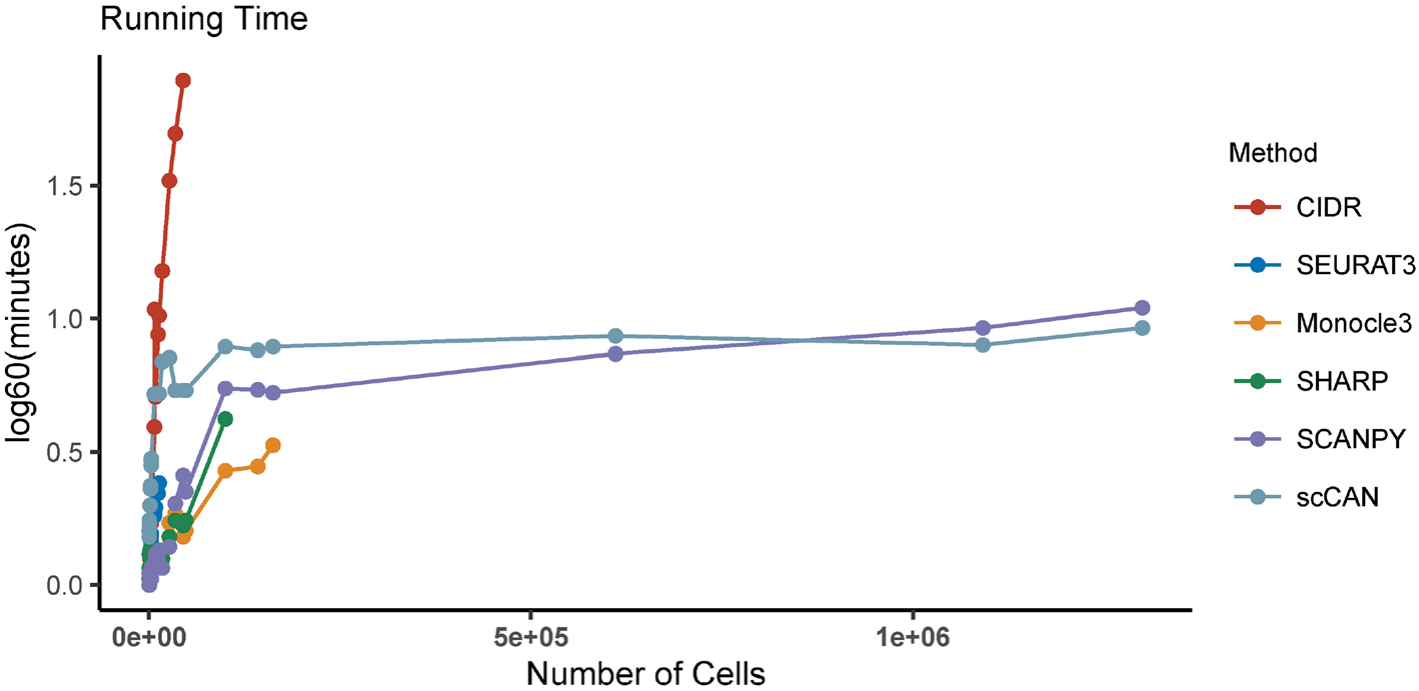
Running time of CIDR, SEURAT3, Monocle3, SHARP, SCANPY, and scCAN for the analysis of 28 real scRNA-seq datasets. The horizontal axis shows the number of cells while the vertical axis shows the running time in the log scale (base 60) of minutes. scCAN and SCANPY are the only two methods that can analyze datasets with more than 200,000 cells.

In order to assess the scalability of the clustering methods, we record the running time that each method uses to analyze the 28 real datasets. Figure 5 shows the running time of the methods with varying numbers of cells. The time complexity of CIDR increases exponentially with respect to sample size. Supplementary Table S7 shows the detailed running time of each method for all 28 datasets. The cell with “NA” indicates out of memory or error. The memory of our machine is limited to 256 GB. scCAN and SCANPY can cluster all datasets in minutes. The scalability of scCAN and SCANPY for big data analysis is shown at Supplementary Section 10 and Supplementary Figures S13 and S14. CIDR, SEURAT3, Monocle3, and SHARP are unable to cluster datasets with more than 48,000, 17,000, 600,000, and 140,000 cells, respectively.

## Conclusion

In this article, we introduce a new clustering method named scCAN that can accurately segregate cells of different types from large-scale scRNA-seq data. The contribution of scCAN is three-fold: (1) effective noise detachment and dimension reduction using a non-negative-kernel and a Stacked Variational Autoencoder, (2) accurate clustering of cells using network fusion and graph-based analysis, and (3) scalable analysis of large number of cells using sampling and k-nearest neighbor. In an extensive analysis using 28 real scRNA-seq datasets and various simulation scenarios, we demonstrate that scCAN outperforms the current state-of-the-art methods, CDIR, SEURAT3, Monocle3, SHARP and SCANPY. The method can: (1) accurate estimate the number of cell types, (2) properly segregate cells of different types, (3) is robust against dropouts, and (4) is able to analyze datasets with more than a million cells in minutes. We also provide a CRAN R package with documentation for users. The tool can be seamlessly embedded into other single-cell analysis pipelines.

## Supplementary Information

Supplementary Information.

## Data Availability

In this manuscript, we analyzed 28 publicly available datasets. The Accession numbers are reported in Supplementary Table S1. The link to each dataset is available in Supplementary Table S2. The scCAN package is available at https://cran.r-project.org/package=scCAN. Processed data and R scripts are available at http://sccan.tinnguyen-lab.com/
