# Supplementary material for: scCAN: single-cell clustering using autoencoder and network fusion
Source: Sci Rep. 2022 Jun 17;12:10267. doi: 10.1038/s41598-022-14218-6 (PMC9206025; doi:10.1038/s41598-022-14218-6)
Supplement: Supplementary file 1 — Supplementary Information. [file 41598_2022_14218_MOESM1_ESM.pdf]

scCAN: Single cell clustering using autoencoder and network fusion

Supplementary Material

Bang Tran[1], Duc Tran[1], Hung Nguyen[1], Seungil Ro[2], and Tin Nguyen[1,*]

[1] Department of Computer Science and Engineering, University of Nevada Reno, Reno, NV 89557, USA
[2] Department of Physiology and Cell Biology, University of Nevada School of Medicine, Reno, NV 89557, USA
[*] tinn@unr.edu

Contents

1 Software packages and settings **3**

2 Data availability **3**

3 Evaluation metrics **6**

3.1 Adjusted Rand index 6

3.2 Adjusted mutual information 6

3.3 V-Measure 7

3.4 Absolute symmetric log-modulus 7

4 Correct estimation of the number of cell types **8**

5 Clustering results of real datasets **9**

6 Visualization of clustering results **13**

7 Comparison of the clustering methods used in Modules 2 and 3 **24**

8 Effects of min-max scaling **26**

9 Rare cell types detection **27**

10 Scalability of scCAN **29**

References **31**

1 Software packages and settings

To compare scCAN with current methods, the following packages are used in the analysis: i) CIDR version 0.1.5 from GitHub (https://github.com/VCCRI/CIDR), ii) SEURAT3 version 3.2.3 from Github (https://github.com/satijalab/seurat/releases/tag/v3.2.3), iii) Monocle3 version 3.0 from Github (https://github.com/cole-trapnell-lab/monocle3), iv) SHARP version 1.1.0 from Github (https://github.com/shibiaowan/SHARP), and SCANPY version 1.4.4 from Anaconda. We carefully follow the instruction and tutorial provided by the authors of each package. We execute each method using default parameters suggested by the authors.

2 Data availability

We downloaded 28 scRNA-seq datasets from public repositories. The datasets Guo, Kanton, Brann, and Miller were downloaded from the European Bioinformatics Institute (https://www.ebi.ac.uk/gxa/sc/experiments/). The datasets Slyper, Zilionis, Orozco, and Kozareva were downloaded from Broad Institute Single Cell Portal (https://singlecell.broadinstitute.org/single_cell). The datasets Montoro, Hrvatin, Darrah, and Cao were downloaded from NCBI[1]. The Brain 1.3M dataset was downloaded from the 10X Genomics website (https://support.10xgenomics.com/single-cell-gene-expression/datasets/1.3.0/1M_neurons). The remaining 15 datasets were downloaded from Hemberg Group's website (https://hemberg-lab.github.io/scRNA.seq.datasets). The Table S1 reports the Accession numbers, and Table S2 shows the specific link to each of the 28 datasets.

Table S1. Description of the 28 single-cell datasets used to assess the performance of scCAN. The first two columns describe the name and tissue while the next five columns show the number of cells, number of cell types, sequencing protocol, accession ID, and references. The first 27 datasets have true cell labels and can be used to assess the accuracy of the clustering methods.

Dataset	Tissue	Size	Class	Protocol	Accession ID	Reference
**1. Pollen**	Human Tissues	301	11	SMARTer	SRP041736	[2]
**2. Patel**	Human Tissues	430	5	Smart-Seq	GSE57872	[3]
**3. Wang**	Human Pancreas	457	7	SMARTer	GSE83139	[4]
**4. Li**	Human Tissues	561	9	SMARTer	GSE81861	[5]
**5. Usoskin**	Mouse Brain	622	4	STRT-Seq	GSE59739	[6]
**6. Camp**	Human Liver	777	7	SMARTer	GSE81252	[7]
**7. Xin**	Human Pancreas	1,600	8	SMARTer	GSE81608	[8]
**8. Muraro**	Human Pancreas	2,126	10	CEL-Seq2	GSE85241	[9]
**9. Segerstolpe**	Human Pancreas	2,209	14	Smart-Seq2	E-MTAB-5061	[10]
**10. Romanov**	Mouse Brain	2,881	7	SMARTer	GSE74672	[11]
**11. Zeisel**	Mouse Brain	3,005	9	STRT-Seq	GSE60361	[12]
**12. Lake**	Human Brain	3,042	16	Fluidigm C1	phs000833.v3.p1	[13]
**13. Montoro**	Human Pancreas	7,193	7	Smart-Seq2	GSE103354	[14]
**14. Guo**	Human Testis	7,416	7	10X Genomics	E-GEOD-134144	[15]
**15. Baron**	Human Pancreas	8,569	14	inDrop	GSE84133	[16]
**16. Chen**	Mouse Brain	12,089	46	Drop-seq	GSE87544	[17]
**17. Slyper**	Human Blood	13,316	8	10X Genomics	SCP345	
**18. Kanton**	Human Brain	17,542	14	Smart-Seq2	E-HCAD-5	[18]
**19. Brann**	Mouse Brain	26,766	46	10X Genomics	E-GEOD-151346	[19]
**20. Zilionis**	Human Lung	34,558	9	inDrop	GSE127465	[20]
**21. Macosko**	Mouse Retina	44,808	12	Drop-seq	GSE63473	[21]
**22. Hrvatin**	Mouse Visual Cortex	48,266	8	inDrop	GSE102827	[22]
**23. Orozco**	Human Eye	100,055	11	10X Genomics	GSE135133	[23]
**24. Miller**	Human Lung	142,523	11	10X Genomics	E-MTAB-8221	[24]
**25. Darrah**	Human Blood	162,490	14	Drop-seq	GSE139598	[25]
**26. Kozareva**	Mouse Cerebellum	611,034	18	10X Genomics	SCP795	[26]
**27. Cao**	Mouse Cerebellum	1,092,000	9	10X Genomics	GSE156793	[27]
**28. Brain 1.3M**	Mouse Brain	1,300,774	NA	10X Genomics	GSE93421	[28]

Table S2. Link to 28 single-cell datasets.

Dataset	Link	Reference
**1. Pollen**	https://hemberg-lab.github.io/scRNA.seq.datasets/human/tissues/#pollen	[2]
**2. Patel**	https://hemberg-lab.github.io/scRNA.seq.datasets/human/tissues/#patel	[3]
**3. Wang**	https://hemberg-lab.github.io/scRNA.seq.datasets/human/pancreas/#wang	[4]
**4. Li**	https://hemberg-lab.github.io/scRNA.seq.datasets/human/brain/#li	[5]
**5. Usoskin**	https://hemberg-lab.github.io/scRNA.seq.datasets/mouse/brain/#usoskin	[6]
**6. Camp**	https://hemberg-lab.github.io/scRNA.seq.datasets/human/liver/	[7]
**7. Xin**	https://hemberg-lab.github.io/scRNA.seq.datasets/human/pancreas/#xin	[8]
**8. Muraro**	https://hemberg-lab.github.io/scRNA.seq.datasets/human/pancreas/#muraro	[9]
**9. Segerstolpe**	https://hemberg-lab.github.io/scRNA.seq.datasets/human/pancreas/#segerstolpe	[10]
**10. Romanov**	https://hemberg-lab.github.io/scRNA.seq.datasets/mouse/brain/#romanov	[11]
**11. Zeisel**	https://hemberg-lab.github.io/scRNA.seq.datasets/mouse/brain/#zeisel	[12]
**12. Lake**	https://hemberg-lab.github.io/scRNA.seq.datasets/human/brain/#lake	[13]
**13. Montoro**	https://www.ncbi.nlm.nih.gov/geo/query/acc.cgi?acc=GSE103354	[14]
**14. Guo**	https://www.ebi.ac.uk/gxa/sc/experiments/E-GEOD-134144/	[15]
**15. Baron**	https://hemberg-lab.github.io/scRNA.seq.datasets/human/pancreas/	[16]
**16. Chen**	https://hemberg-lab.github.io/scRNA.seq.datasets/mouse/brain/#chen	[17]
**17. Slyper**	https://singlecell.broadinstitute.org/single_cell/study/SCP345/	
**18. Kanton**	https://www.ebi.ac.uk/gxa/sc/experiments/E-HCAD-5/	[18]
**19. Brann**	https://www.ebi.ac.uk/gxa/sc/experiments/E-GEOD-151346/	[19]
**20. Zilionis**	https://singlecell.broadinstitute.org/single_cell/study/SCP739/	[20]
**21. Macosko**	https://hemberg-lab.github.io/scRNA.seq.datasets/mouse/retina/	[21]
**22. Hrvatin**	https://www.ncbi.nlm.nih.gov/geo/query/acc.cgi?acc=GSE102827	[22]
**23. Orozco**	https://singlecell.broadinstitute.org/single_cell/study/SCP484/	[23]
**24. Miller**	https://www.ebi.ac.uk/gxa/sc/experiments/E-MTAB-8221/	[24]
**25. Darrah**	https://www.ncbi.nlm.nih.gov/geo/query/acc.cgi?acc=GSE139598	[25]
**26. Kozareva**	https://singlecell.broadinstitute.org/single_cell/study/SCP795/	[26]
**27. Cao**	https://www.ncbi.nlm.nih.gov/geo/query/acc.cgi?acc=GSE156793	[27]
**28. Brain 1.3M**	https://support.10xgenomics.com/single-cell-gene-expression/datasets/1.3.0/1M_neurons	[28]

3 Evaluation metrics

We use three different metrics for comparing the obtained partitions with the known cell types: adjusted Rand index (ARI)[29], adjusted mutual information (AMI)[30], and V-measure[31]. To evaluate the capability of each method in predicting the true number of clusters, we use absolute log-modulus[32].

3.1 Adjusted Rand index

Rand index (RI) evaluates the similarity between predicted clusters and true cell types. Given P as a set of clusters and Q is a set of true cell types then RI is calculated as:

$$RI = \frac{t+u}{t+u+v+s} = \frac{t+u}{\binom{N}{2}} \tag{1}$$

where t is the number of pairs belonging to the same cell type in Q and are grouped together in the same cluster in P, u is the number of pairs of different cell types in Q and are grouped to different clusters in P, v is the number of pairs of the same cell types in Q and are grouped to different clusters in P, s is the number of pairs in different cell types in Q and are grouped together in the same cluster in P, N is the total number of cells, and $\binom{N}{2}$ is the number of possible pairs. In brief, RI measures the ratio of pairs that are clustered in the same way (either together or different) from two partitions (e.g. 0.80 means 80% of pairs are grouped in the same way). The Adjusted Rand Index (ARI)[29] is the corrected-for-chance version of the Rand Index. The ARI values ranged from -1 to 1 in which 0 indicates a random grouping. The ARI score is calculated as :

$$ARI = \frac{RI - exptected\_RI}{max(RI) - expected\_RI} \tag{2}$$

3.2 Adjusted mutual information

Adjusted mutual information (AMI) is an adjustment of the mutual information (MI) score to account for random partitioning. It accounts for the fact that the MI is generally higher for two clusters with a larger number of clusters, regardless of whether there is actually more information shared. The calculation of AMI is presented as follows:

Given a dataset of n cells with true partition $X = \{X_1, X_2, ..., X_R\}$ of R clusters and predicted partition $Y = \{Y_1, Y_2, ..., Y_C\}$ of C clusters. The mutual information of cluster overlap between X and Y can be summarized as a contingency table $M_{R\times C} = [n_{ij}]$, where $i = 1...R$, $j = 1...C$, and n_{ij} represents the number of common data point falls into cluster X_i is $p(i) = \frac{|x_i|}{n}$. The entropy associated with the clustering X is calculated as follows:

$$H(X) = \sum_{i=1}^{R} P(i) log P(i) \tag{3}$$

$H(X)$ gives outputs as non-negative values where 0 indicates that there is one cluster in the dataset. The mutual information (MI) between two clusters X and Y is calculated as follows:

$$MI(X,Y) = \sum_{i=1}^{R}\sum_{j=1}^{C} P(i,j) log \frac{P(i,j)}{P(i)P(j)} \frac{n_{ij}}{n} \tag{4}$$

where $P(i,j)$ is the cell that is classified to both clusters X_i in X and Y_j in Y. $P(i,j)$ is calculated as follows:

$$P(i,j) = \frac{|X_i \cap Y_j|}{n} \tag{5}$$

MI gives outputs as non-negative values bounded by the entropies $H(X)$ and $H(Y)$ and 0 indicates that there is no cell classified to the same cluster. To correct for the fact that two random clusterings do not give a constant value, and tends to be larger when the two partitions have a larger number of clusters. Therefore, *AM*I is defined as follows:

$$AMI(X,Y) = \frac{MI(X,Y) - E\{MI(X,Y)\}}{max\{H(X),H(Y)\} - E\{MI(X,Y)\}} \tag{6}$$

where $E\{MI(X,Y)\}$ is the expected mutual information between two random clusterings. The *AMI* takes value between 0 and 1 where 0 stands for random clustering and 1 represents a perfect partition.

3.3 V-Measure

V-Measure is the harmonic mean between two measures: homogeneity and completeness. Homogeneous clustering is when each cluster has data points belonging to the same class. Complete clustering is when all dat a points belonging to the same class are clustered into the same cluster. Given a set of classes $C = \{C_1, C_2, ..., C_l\}$, a set of cluster $K = \{K_1, K_2, ..., K_m\}$ and the conditional entropy of the class distribution given the identified clustering is computed as $H(C|K)$. The homogeneity is defined as follows:

$$h = \begin{cases} 1 & if H(C|K) = 0 \\ 1 - \frac{H(C|K)}{H(C)} & otherwise \end{cases} \tag{7}$$

The completeness is symmetrical to homogeneity. To measure the completeness, the distribution of cluster assignments within each class is assessed. In a perfect clustering, each of these distributions will be completely skewed to a single cluster. Given the homogeneity h and completeness c, the V-measure is computed as the weighted harmonic mean β between homogeneity and completeness:

$$V - measure = \frac{1 + \beta * h * c)}{(\beta * h) + c} \tag{8}$$

if β is greater than 1, completeness is weighted more strongly in the calculation. If β is less than 1, homogeneity is weighted more strongly. Since the computations of homogeneity, completeness and V-measure are completely independent of the number of classes, the number of clusters, the size of the dataset and the clustering algorithm, these measures can be employed for evaluating any clustering solution.

3.4 Absolute symmetric log-modulus

To evaluate the accuracy of methods in estimating the correct number of clusters, we used absolute symmetric log-modulus[32] transformation defined as follows:

$$L(x) = |sign(x) * log10(|x| + 1)| \tag{9}$$

where x is the difference between the estimated number of clusters and the true number of cell types in a given dataset. The higher values of absolute log-modulus transformation mean the number of estimated clusters is more different from the number of true cell types. x equals to zero denotes the perfect estimation.

4 Correct estimation of the number of cell types

Table S3. Estimation of the number of cell types of CIDR, SEURAT3, Monocle3, SHARP, SCANPY, and scCAN on 27 single-cell datasets measured by absolute log-modulus values. Cells with NA values indicate that the method was not able to analyze the dataset (crashed or out-of-memory). The average absolute log-modulus value of scCAN is 0.59, which are smaller than the rest.

Datasets	CIDR	Seurat3	Monocle3	SHARP	SCANPY	scCAN
**Pollen**	0.60	0.30	0.30	0.70	0.60	0.60
**Patel**	0.30	0.30	0.60	0.60	0.70	0.00
**Wang**	0.00	0.00	0.78	0.60	0.60	0.48
**Li**	0.78	0.48	0.30	1.00	0.00	0.48
**Usoskin**	0.30	0.48	0.90	0.30	1.04	0.00
**Camp**	0.60	0.48	1.00	0.00	0.78	0.78
**Xin**	0.85	0.30	1.08	0.48	0.60	0.60
**Muraro**	0.70	0.90	1.26	0.48	1.18	0.70
**Segerstolpe**	0.60	0.95	1.51	0.00	1.41	0.70
**Romanov**	0.00	0.90	1.32	0.48	1.26	0.30
**Zeisel**	0.70	0.95	1.40	0.78	1.30	0.30
**Lake**	0.70	0.00	1.40	0.95	1.04	0.30
**Montoro**	0.78	0.30	1.41	1.04	0.60	0.90
**Guo**	0.30	0.78	1.52	0.85	1.23	0.30
**Baron**	0.70	0.70	1.57	0.85	1.15	0.30
**Chen**	1.00	0.30	1.58	0.90	1.11	0.78
**Slyper**	0.60	0.85	1.58	0.48	1.08	0.48
**Kanton**	0.78	0.30	1.66	1.08	0.85	0.78
**Brann**	1.64	1.51	1.20	1.64	1.51	1.60
**Zilionis**	0.70	1.00	1.68	0.60	1.08	0.48
**Macosko**	0.60	0.90	1.78	0.78	0.78	0.70
**Hrvatin**	NA	0.78	1.83	0.90	1.00	0.48
**Orozco**	NA	1.28	2.07	1.08	1.61	0.85
**Miller**	NA	NA	2.03	NA	1.32	0.95
**Darrah**	NA	NA	1.93	NA	1.36	0.48
**Kozareva**	NA	NA	NA	NA	0.85	0.95
**Cao**	NA	NA	NA	NA	1.04	0.60
**Mean**	0.63	0.64	1.35	0.72	1.00	0.59

5 Clustering results of real datasets

Table S4. Performance of CIDR, SEURAT3, Monocle3, SHARP, SCANPY, and scCAN on 27 single-cell datasets measured by Adjusted Rand Index (ARI). Cells with NA values indicate that the method was not able to analyze the dataset (crashed or out-of-memory). Cells highlighted in bold have the highest ARI values. The average ARI of scCAN is 0.81, which is much higher than the rest (SEURAT3 is the second best with an average ARI of 0.55). In addition, scCAN has the highest ARI values in all but three datasets (Camp, Montoro and Hrvatin).

Dataset	#Cells	CIDR	SEURAT3	Monocle3	SHARP	SCANPY	scCAN
**Pollen**	301	0.90	0.73	0.82	0.09	0.77	**0.92**
**Patel**	430	0.45	0.82	0.26	0.09	0.66	**0.86**
**Wang**	457	0.63	0.56	0.28	0.41	0.62	**0.83**
**Li**	561	0.62	0.84	0.77	0.19	0.81	**0.94**
**Usoskin**	622	0.82	0.56	0.35	0.07	0.34	**0.93**
**Camp**	777	0.61	**0.65**	0.55	0.44	0.61	0.61
**Xin**	1,600	0.57	0.50	0.15	0.56	0.29	**0.98**
**Muraro**	2,126	0.22	0.64	0.30	0.31	0.43	**0.91**
**Segerstolpe**	2,209	0.37	0.60	0.20	0.33	0.31	**0.95**
**Romanov**	2,881	0.32	0.48	0.19	0.59	0.30	**0.63**
**Zeisel**	3,005	0.37	0.50	0.24	0.46	0.32	**0.86**
**Lake**	3,042	0.47	0.51	0.23	0.21	0.43	**0.58**
**Montoro**	7,193	0.30	0.24	0.08	**0.80**	0.20	0.70
**Guo**	7,416	0.75	0.62	0.23	0.24	0.46	**0.86**
**Baron**	8,569	0.73	0.56	0.21	0.36	0.46	**0.94**
**Chen**	12,089	0.36	0.69	0.25	0.59	0.62	**0.72**
**Slyper**	13,316	0.63	0.24	0.06	0.39	0.26	**0.67**
**Kanton**	17,542	0.47	0.40	0.13	0.31	0.47	**0.67**
**Brann**	26,766	0.12	0.32	0.06	0.76	0.32	**0.86**
**Zilionis**	34,558	0.53	NA	0.12	0.37	0.38	**0.89**
**Macosko**	44,808	0.17	NA	0.04	0.71	0.23	**0.85**
**Hrvatin**	48,266	NA	NA	0.13	**0.92**	0.57	0.78
**Orozco**	100,055	NA	NA	0.04	0.20	0.22	**0.77**
**Miller**	142,523	NA	NA	0.04	NA	0.16	**0.90**
**Darrah**	162,490	NA	NA	0.02	NA	0.08	**0.47**
**Kozareva**	611,034	NA	NA	NA	NA	0.12	**1.00**
**Cao**	1,092,000	NA	NA	NA	NA	0.48	**0.89**
**Mean**		0.50	0.55	0.23	0.41	0.40	**0.81**

Table S5. Performance of CIDR, SEURAT3, Monocle3, SHARP, SCANPY, and scCAN on 27 single-cell datasets measured by Adjusted Mutual Information (AMI). Cells with NA values indicate that the method was not able to analyze the dataset (crashed or out-of-memory). Cells highlighted in bold have the highest AMI values. The average AMI of scCAN is 0.77, which is much higher than the rest (SEURAT3 is the second best with an average AMI of 0.64). In addition, scCAN has the highest AMI values in all but four datasets (Camp, Montoro, Chen and Hrvatin).

Dataset	#Cells	CIDR	SEURAT3	Monocle3	SHARP	SCANPY	scCAN
**Pollen**	301	0.91	0.80	0.84	0.20	0.88	**0.93**
**Patel**	430	0.55	0.77	0.29	0.16	0.64	**0.84**
**Wang**	457	0.66	0.60	0.42	0.40	0.64	**0.75**
**Li**	561	0.69	0.88	0.84	0.27	0.84	**0.95**
**Usoskin**	622	0.76	0.61	0.48	0.19	0.48	**0.88**
**Camp**	777	0.72	**0.77**	0.67	0.59	0.72	0.72
**Xin**	1,600	0.51	0.57	0.35	0.50	0.44	**0.91**
**Muraro**	2,126	0.41	0.72	0.53	0.31	0.60	**0.87**
**Segerstolpe**	2,209	0.42	0.72	0.47	0.33	0.55	**0.88**
**Romanov**	2,881	0.33	0.55	0.37	0.52	0.44	**0.61**
**Zeisel**	3,005	0.38	0.58	0.46	0.46	0.50	**0.81**
**Lake**	3,042	0.47	0.65	0.53	0.22	0.67	**0.74**
**Montoro**	7,193	0.35	0.35	0.25	**0.64**	0.33	0.58
**Guo**	7,416	0.76	0.71	0.49	0.51	0.59	**0.87**
**Baron**	8,569	0.65	0.69	0.49	0.40	0.64	**0.87**
**Chen**	12,089	0.37	0.75	0.59	0.52	**0.75**	0.55
**Slyper**	13,316	0.68	0.46	0.31	0.30	0.46	**0.73**
**Kanton**	17,542	0.49	0.53	0.39	0.30	0.57	**0.64**
**Brann**	26,766	0.13	0.53	0.33	0.52	0.54	**0.72**
**Zilionis**	34,558	0.50	NA	0.40	0.41	0.53	**0.84**
**Macosko**	44,808	0.27	NA	0.26	0.41	0.42	**0.66**
**Hrvatin**	48,266	NA	NA	0.41	**0.87**	0.64	0.76
**Orozco**	100,055	NA	NA	0.29	0.32	0.43	**0.65**
**Miller**	142,523	NA	NA	0.23	NA	0.33	**0.82**
**Darrah**	162,490	NA	NA	0.19	NA	0.25	**0.53**
**Kozareva**	611,034	NA	NA	NA	NA	0.39	**0.94**
**Cao**	1,092,000	NA	NA	NA	NA	0.61	**0.84**
**Mean**		0.52	0.64	0.43	0.41	0.55	**0.77**

Table S6. Performance of CIDR, SEURAT3, Monocle3, SHARP, SCANPY, and scCAN on 27 single-cell datasets measured by V-measure. Cells with NA values indicate that the method was not able to analyze the dataset (crashed or out-of-memory). Cells highlighted in bold have the highest V-measure values. The average V-measure of scCAN is 0.81, which is much higher than the rest (SEURAT3 is the second best with an average V-measure of 0.72). In addition, scCAN has the highest V-measure values in all but four datasets (Romanov, Montoro, Chen and Kanton).

Dataset	#Cells	CIDR	SEURAT3	Monocle3	SHARP	SCANPY	scCAN
**Pollen**	301	0.94	0.89	0.91	0.33	0.91	**0.96**
**Patel**	430	0.57	0.79	0.33	0.26	0.72	**0.84**
**Wang**	457	0.71	0.65	0.52	0.53	0.72	**0.81**
**Li**	561	0.77	0.89	0.90	0.41	0.87	**0.96**
**Usoskin**	622	0.80	0.71	0.62	0.23	0.63	**0.93**
**Camp**	777	0.79	**0.82**	0.79	0.66	**0.82**	**0.82**
**Xin**	1,600	0.55	0.68	0.50	0.50	0.58	**0.92**
**Muraro**	2,126	0.43	0.79	0.66	0.46	0.72	**0.87**
**Segerstolpe**	2,209	0.45	0.77	0.62	0.42	0.69	**0.92**
**Romanov**	2,881	0.34	**0.66**	0.49	0.56	0.58	0.62
**Zeisel**	3,005	0.47	0.67	0.60	0.59	0.63	**0.82**
**Lake**	3,042	0.54	0.69	0.63	0.35	0.73	**0.75**
**Montoro**	7,193	0.46	0.49	0.38	**0.70**	0.47	0.65
**Guo**	7,416	0.79	0.81	0.65	0.52	0.73	**0.89**
**Baron**	8,569	0.72	0.77	0.65	0.55	0.76	**0.89**
**Chen**	12,089	0.42	**0.78**	0.69	0.65	0.77	0.60
**Slyper**	13,316	0.70	0.59	0.45	0.42	0.59	**0.73**
**Kanton**	17,542	0.49	0.60	0.52	0.41	**0.65**	0.64
**Brann**	26,766	0.16	0.64	0.48	0.65	0.65	**0.80**
**Zilionis**	34,558	0.58	NA	0.56	0.52	0.65	**0.89**
**Macosko**	44,808	0.33	NA	0.41	0.49	0.56	**0.70**
**Hrvatin**	48,266	NA	NA	0.58	**0.92**	0.78	0.82
**Orozco**	100,055	NA	NA	0.44	0.41	0.60	**0.75**
**Miller**	142,523	NA	NA	0.37	NA	0.49	**0.88**
**Darrah**	162,490	NA	NA	0.32	NA	0.39	**0.63**
**Kozareva**	611,034	NA	NA	NA	NA	0.56	**0.96**
**Cao**	1,092,000	NA	NA	NA	NA	0.74	**0.90**
**Mean**		0.57	0.72	0.56	0.50	0.67	**0.81**

Table S7. The running time of CIDR, SEURAT3, Monocle3, SHARP, SCANPY, and scCAN on 28 single-cell datasets measured in minutes. Cells with NA values indicate that the method was not able to analyze the dataset (crashed or out-of-memory).

Dataset	#Cells	CIDR	SEURAT3	Monocle3	SHARP	SCANPY	scCAN
**Pollen**	301	0.0	0.2	0.1	0.1	0.0	1.3
**Patel**	430	0.0	0.2	0.1	0.6	0.0	1.1
**Wang**	457	0.1	0.3	0.2	0.1	0.0	1.3
**Li**	561	0.1	0.2	0.2	0.1	0.2	1.7
**Usoskin**	622	0.1	0.2	0.1	0.6	0.0	1.4
**Camp**	777	0.1	0.2	0.1	0.1	0.0	1.6
**Xin**	1,600	0.5	0.6	0.2	0.7	0.2	2.4
**Muraro**	2,126	0.5	0.6	0.2	0.3	0.1	3.4
**Segerstolpe**	2,209	0.7	0.6	0.2	0.3	0.1	3.6
**Romanov**	2,881	1.1	0.8	0.2	0.4	0.2	5.3
**Zeisel**	3,005	1.2	0.9	0.2	0.5	0.1	5.8
**Lake**	3,042	1.6	1.2	0.4	0.5	0.3	6.0
**Montoro**	7,193	10.4	1.9	0.3	0.3	0.4	17.9
**Guo**	7,416	68.1	2.0	0.4	0.7	0.3	17.9
**Baron**	8,569	17.1	2.3	0.4	0.4	0.6	17.9
**Chen**	12,089	45.9	3.1	0.4	0.4	0.5	17.9
**Slyper**	13,316	61.9	3.8	0.3	0.5	0.7	17.8
**Kanton**	17,542	123.5	NA	0.7	1.1	0.3	17.9
**Brann**	26,766	501.1	NA	1.6	1.7	0.8	17.9
**Zilionis**	34,558	1037.3	NA	1.0	1.5	3.5	18.5
**Macosko**	44,808	2353.5	NA	1.1	1.6	4.4	18.5
**Hrvatin**	48,266	NA	NA	1.3	1.7	3.2	18.6
**Orozco**	100,055	NA	NA	4.8	11.9	19.6	37.6
**Miller**	142,523	NA	NA	5.2	NA	19.2	36.0
**Darrah**	162,490	NA	NA	7.6	NA	18.3	37.9
**Kozareva**	611,034	NA	NA	NA	NA	33.9	45.0
**Cao**	1,092,000	NA	NA	NA	NA	51.0	39.0
**Brain 1.3M**	1,300,774	NA	NA	NA	NA	70.0	51.0

6 Visualization of clustering results

We plotted both the original and new cluster annotations in the original t-SNE/UMAP plots. We also quantified the correlation between the two annotations (true cell types and clustering) using Rand index (RI). RI measures the agreement between a cluster annotation and the true cell types. In short, $RI = (a+b)/\binom{N}{2}$ where a is the number of pairs that belong to the same true cell type and are clustered together, b is the number of pairs that belong to different true cell types and are not clustered together, and $\binom{N}{2}$ is the number of possible pairs that can be formed from the N cells. Intuitively, RI is the fraction of pairs that are grouped in the same way (either together or not) in the two annotations compared (e.g. 0.9 means 90% of pairs are grouped in the same way).

Figures S1–S5 show the annotations on the original t-SNE plots. For each dataset, we plotted the two annotations side-by-side: the left panel shows the original annotation whereas the right panel shows the new cluster annotation. Figures S6–S10 shows the annotations on the original UMAP plots. For each dataset, we quantified the similarity between the two annotations using Rand index (RI). An RI of 1 indicates the ideal case in which the two annotations are identical. The average RI across all datasets is 0.93. This indicates a strong correlation between the two annotations on the original t-SNE/UMAP plots.

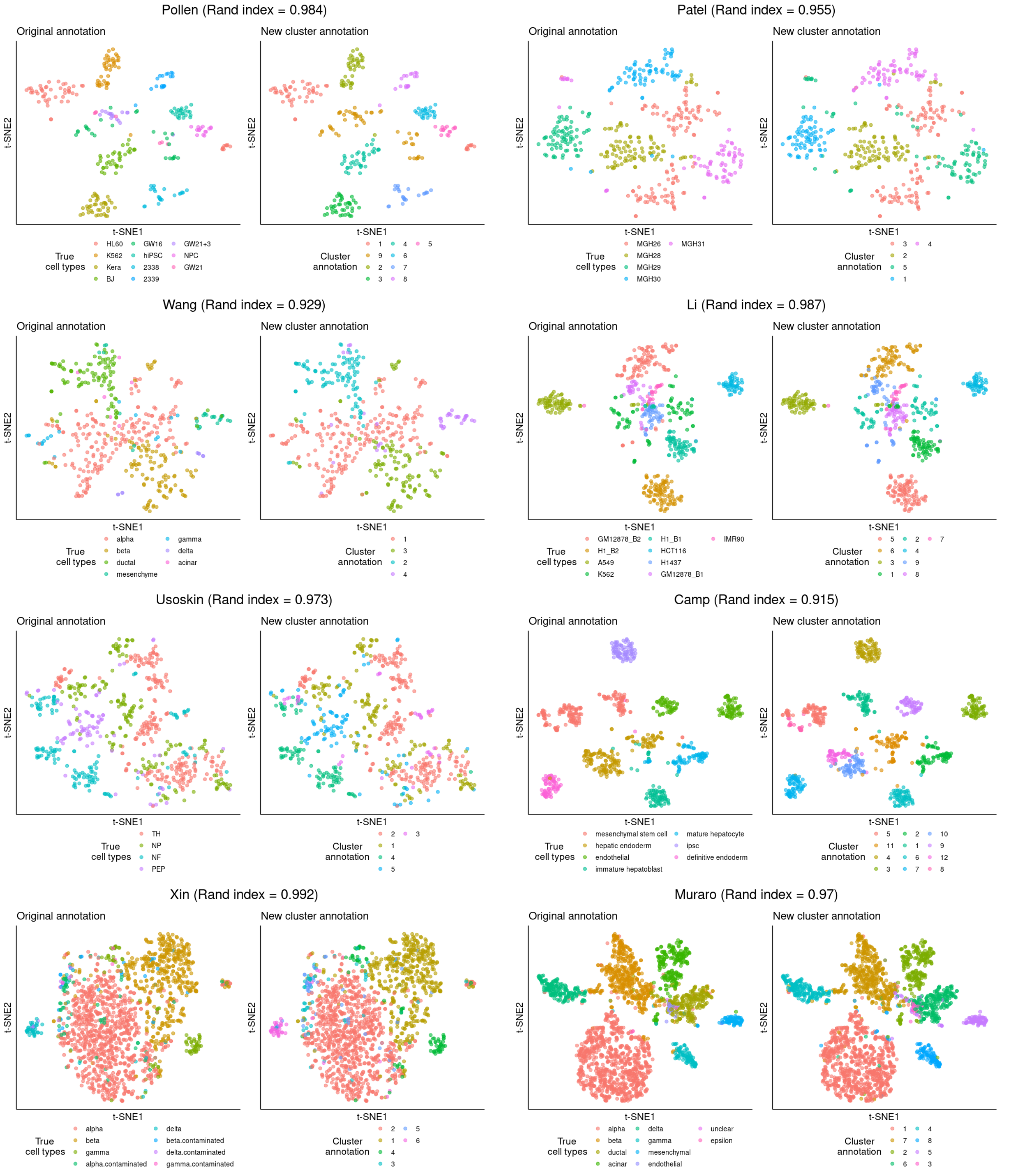

Figure S1. Visualization of the Pollen, Patel, Wang, Li, Usoskin, Camp, Xin and Muraro raw datasets (top to bottom) using t-SNE. For each dataset, the left panels shows t-SNE plot with original labels and the righ panel shows t-SNE plot with cluster annotations identified by scCAN. Different colors codes indicate different cell types and clusters. For each dataset, the Rand index (RI) quantifies the correlation between the original annotation and the new cluster annotation. RI of 1 indicates the ideal case in which the two annotations are identical.

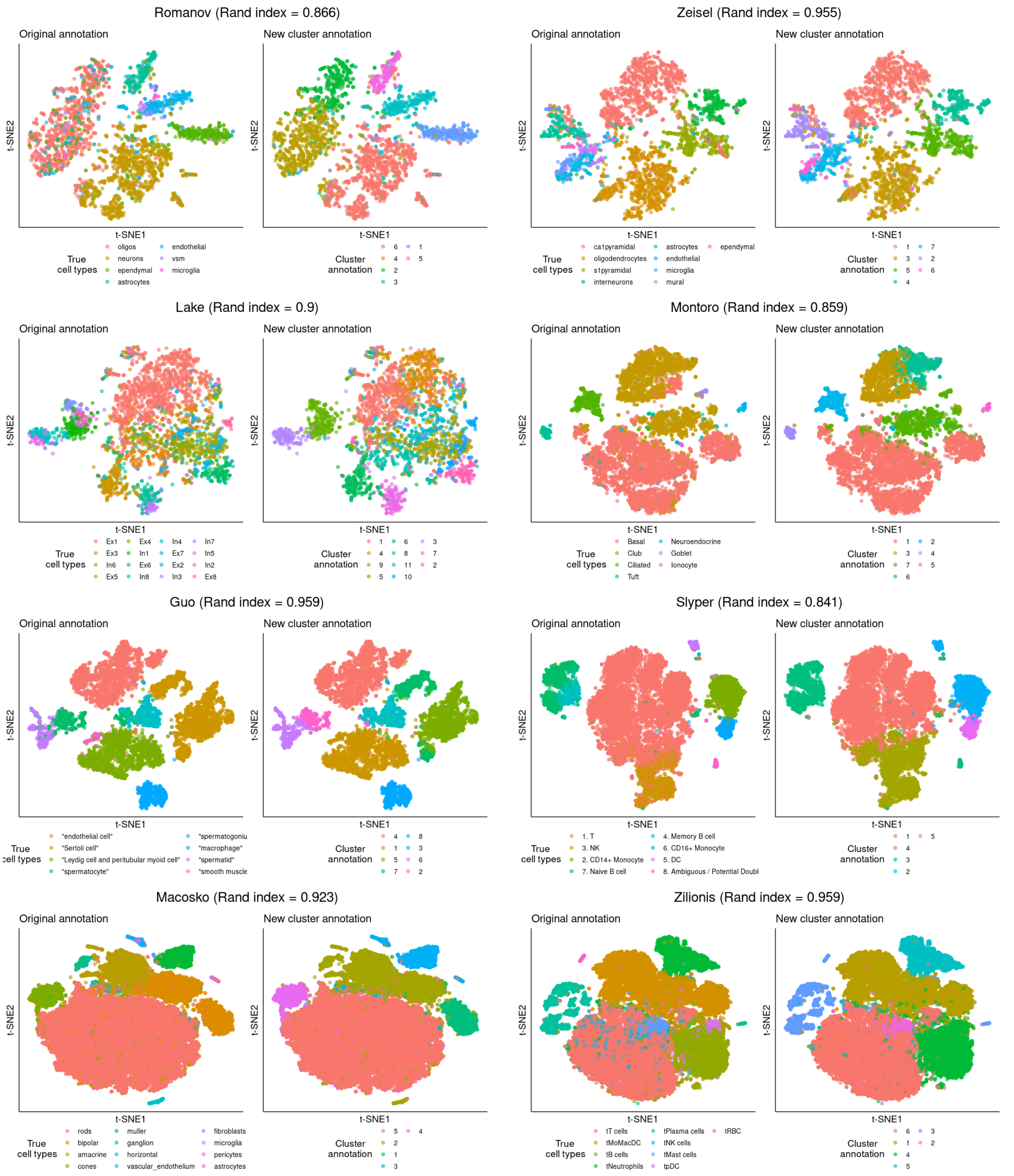

Figure S2. Visualization of the Romanov, Zeisel, Lake, Montoro, Guo, Slyper, Macosko and Zilionis raw datasets (top to bottom) using t-SNE. For each dataset, the left panels shows t-SNE plot with original labels and the righ panel shows t-SNE plot with cluster annotations identified by scCAN. Different colors codes indicate different cell types and clusters.

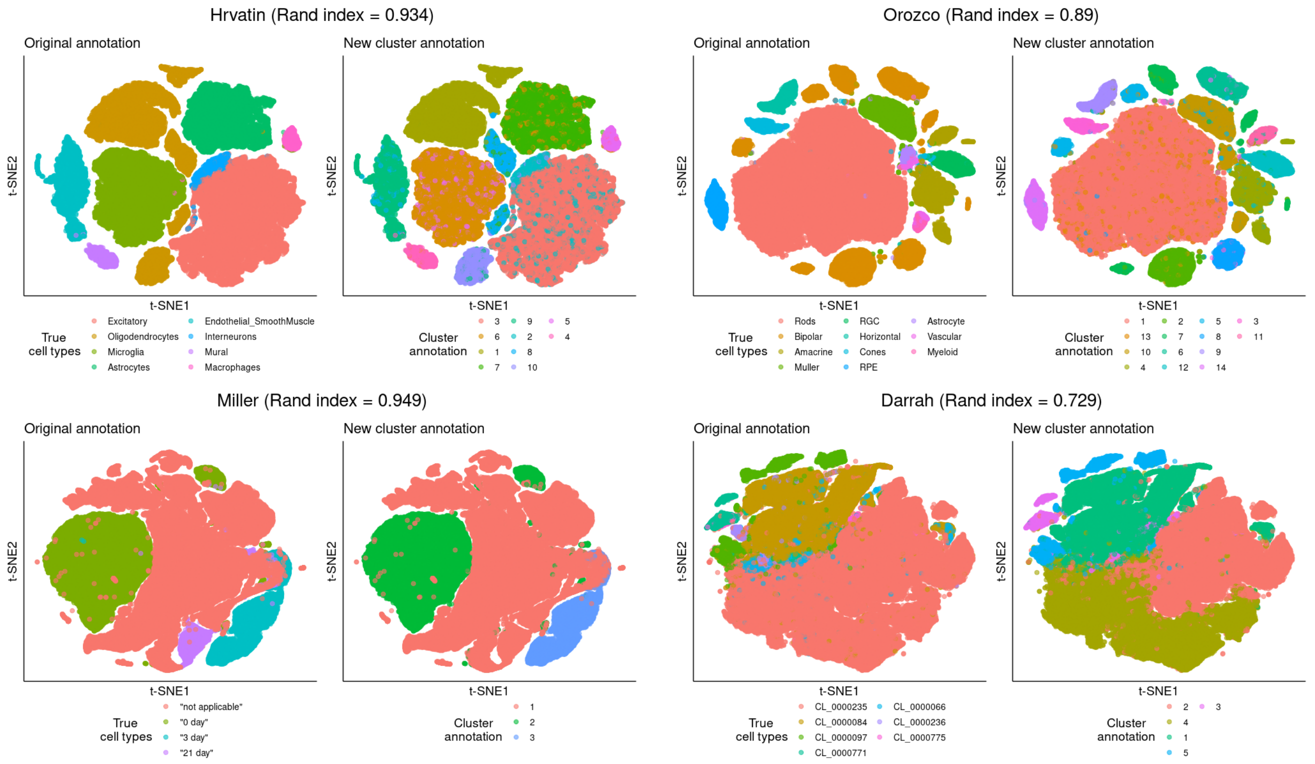

Figure S3. Visualization of the Hrvatin, Orozco, Miller and Darrah raw datasets (top to bottom) using t-SNE. For each dataset, the left panels shows t-SNE plot with original labels and the righ panel shows t-SNE plot with cluster annotations identified by scCAN. Different colors codes indicate different cell types and clusters.

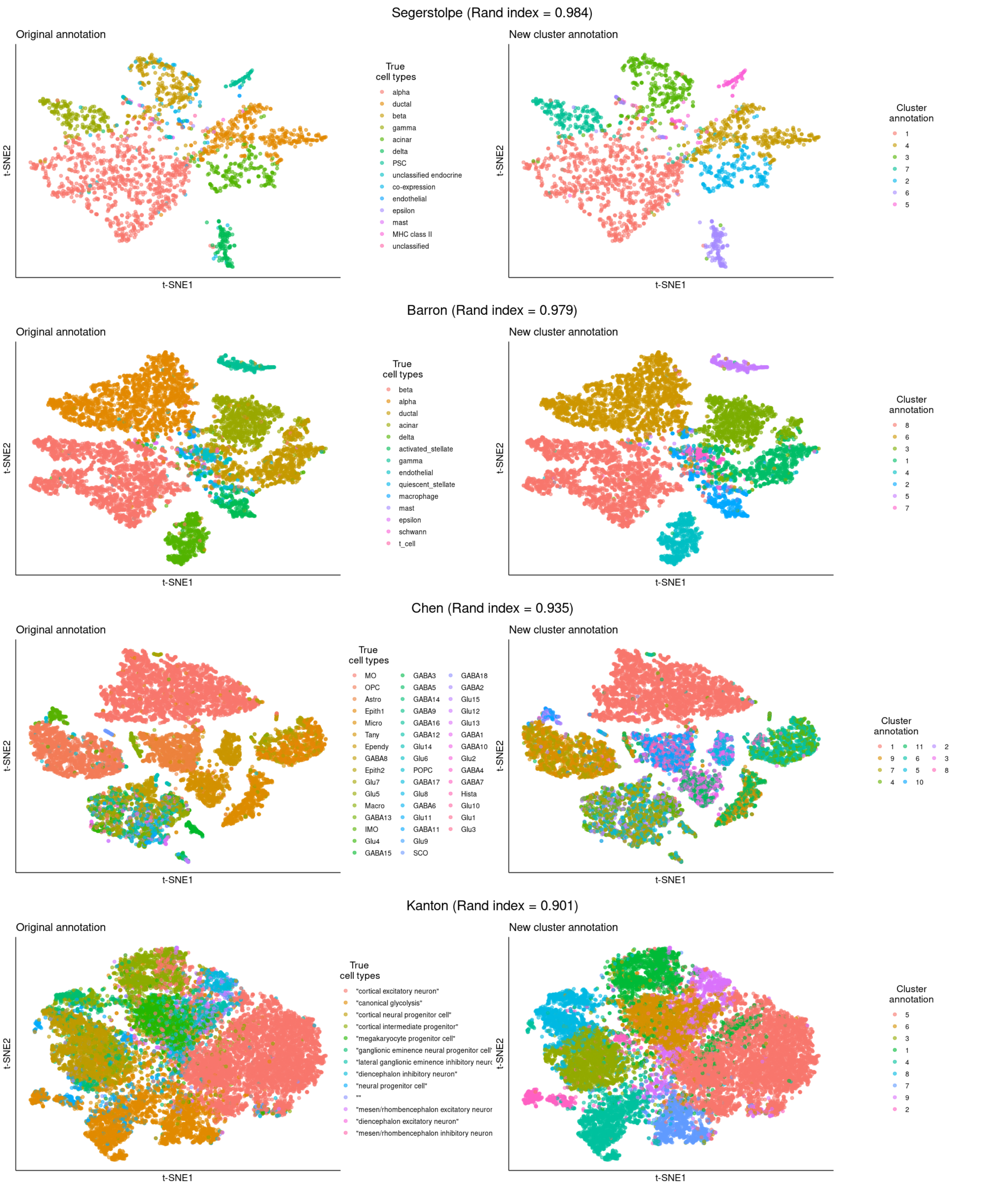

Figure S4. Visualization of the Segerstolpe, Baron, Chen, and Kanton raw datasets (top to bottom) using t-SNE. For each dataset, the left panels shows t-SNE plot with original labels and the righ panel shows t-SNE plot with cluster annotations identified by scCAN. Different colors codes indicate different cell types and clusters.

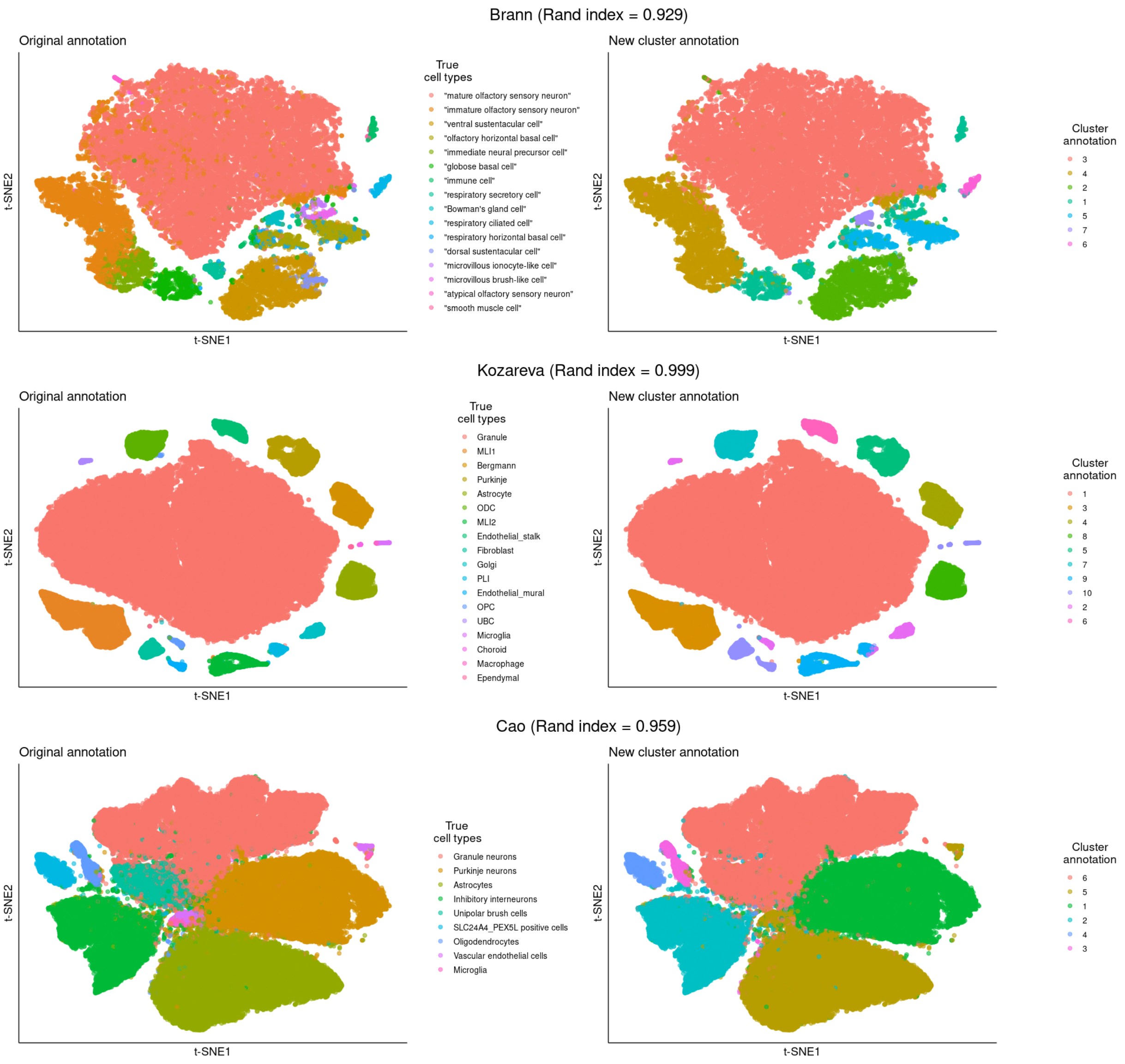

Figure S5. Visualization of the Brann, Kozareva, and Cao raw datasets (top to bottom) using t-SNE. For each dataset, the left panels shows t-SNE plot with original labels and the righ panel shows t-SNE plot with cluster annotations identified by scCAN. Different colors codes indicate different cell types and clusters.

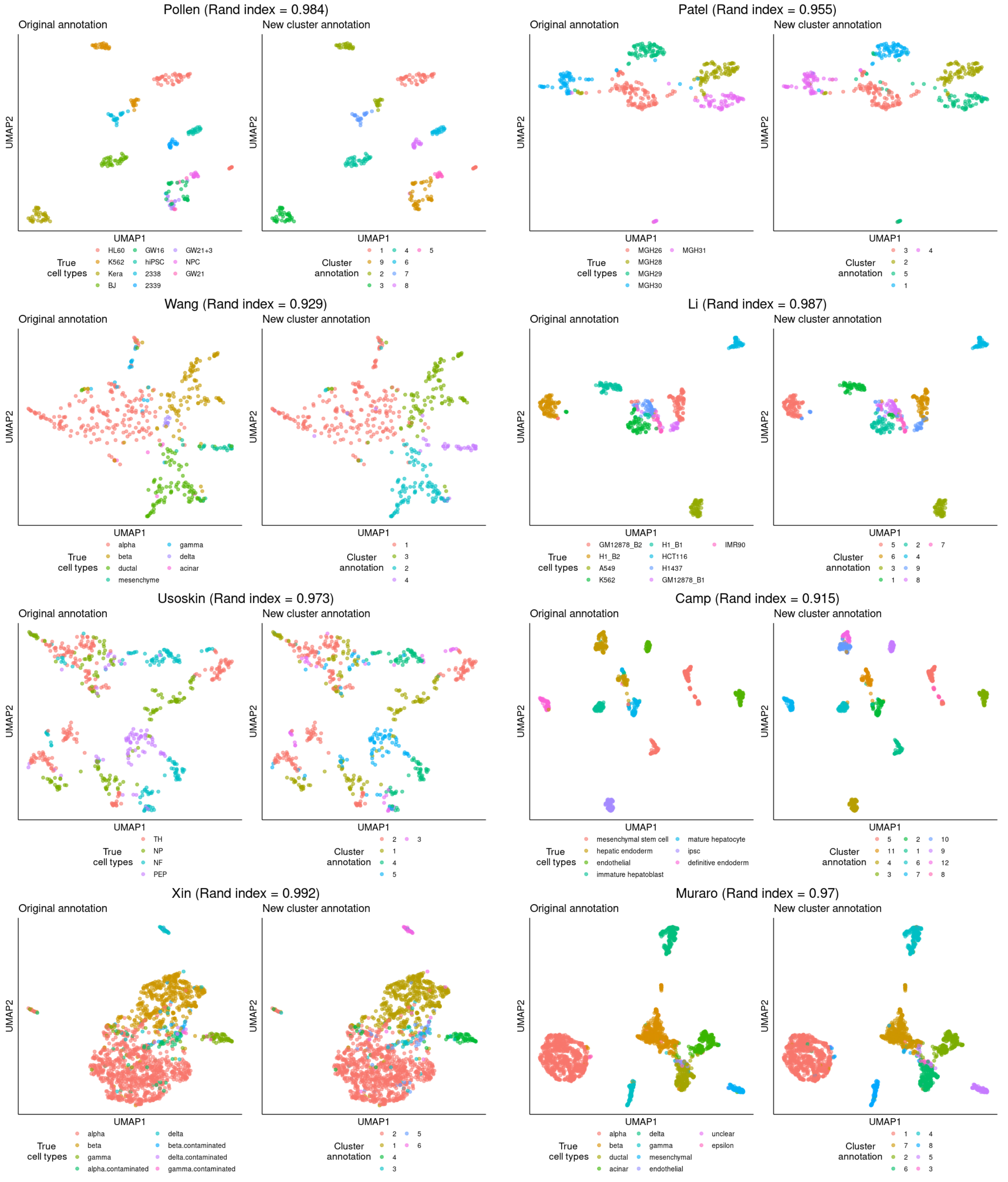

Figure S6. Visualization of the Pollen, Patel, Wang, Li, Usoskin, Camp, Xin and Muraro raw datasets (top to bottom) using UMAP. For each dataset, the left panels shows UMAP plot with original labels and the righ panel shows UMAP plot with cluster annotations identified by scCAN. Different colors codes indicate different cell types and clusters.

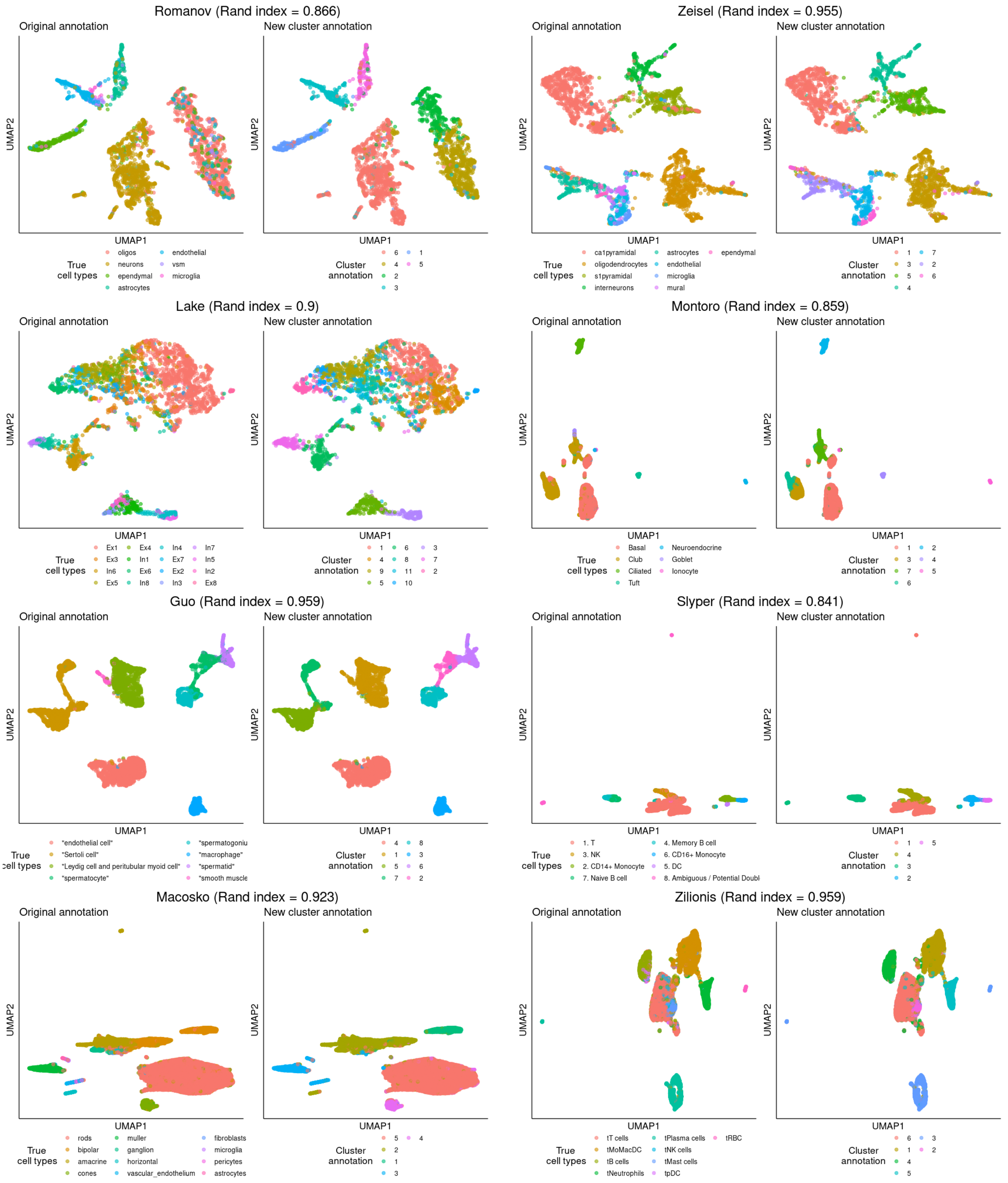

Figure S7. Visualization of the Romanov, Zeisel, Lake, Montoro, Guo, Slyper, Macosko and Zilionis raw datasets (top to bottom) using UMAP. For each dataset, the left panels shows UMAP plot with original labels and the righ panel shows UMAP plot with cluster annotations identified by scCAN. Different colors codes indicate different cell types and clusters.

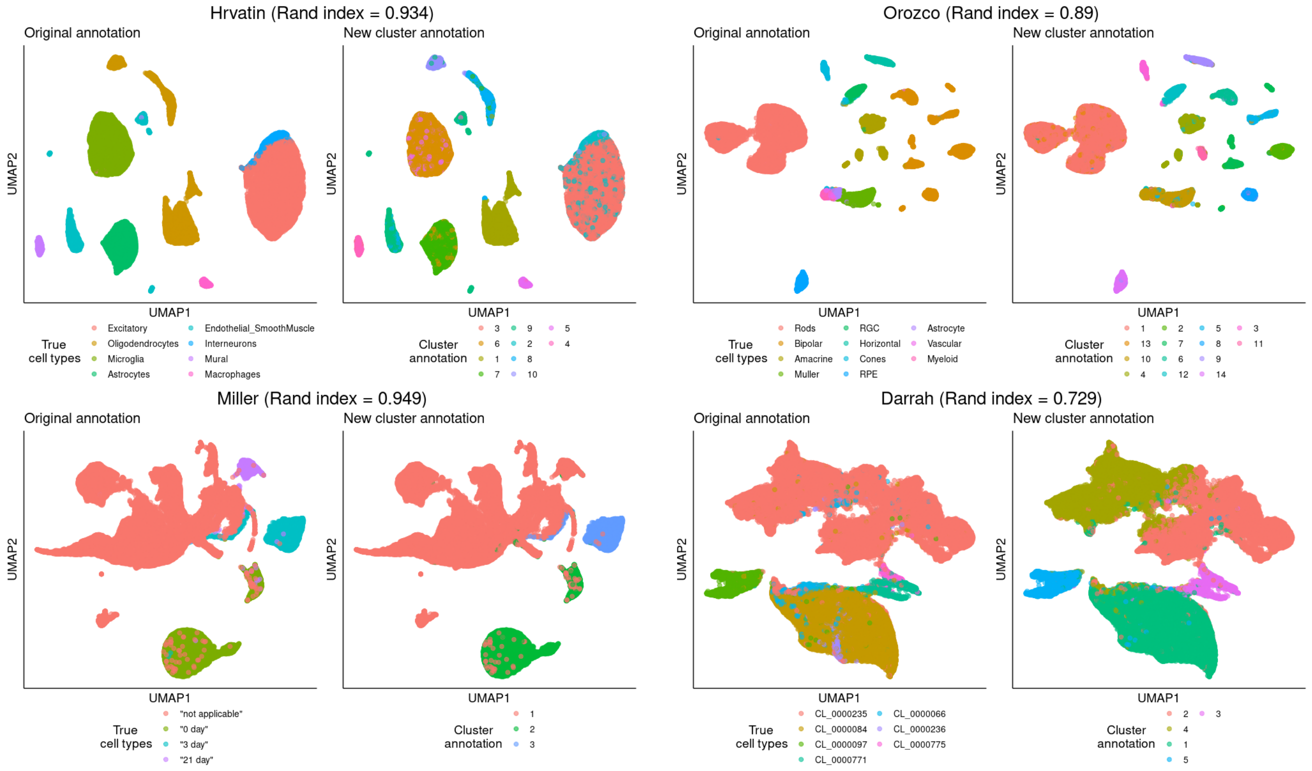

Figure S8. Visualization of the Hrvatin, Orozco, Miller and Darrah raw datasets (top to bottom) using UMAP. For each dataset, the left panels shows UMAP plot with original labels and the righ panel shows UMAP plot with cluster annotations identified by scCAN. Different colors codes indicate different cell types and clusters.

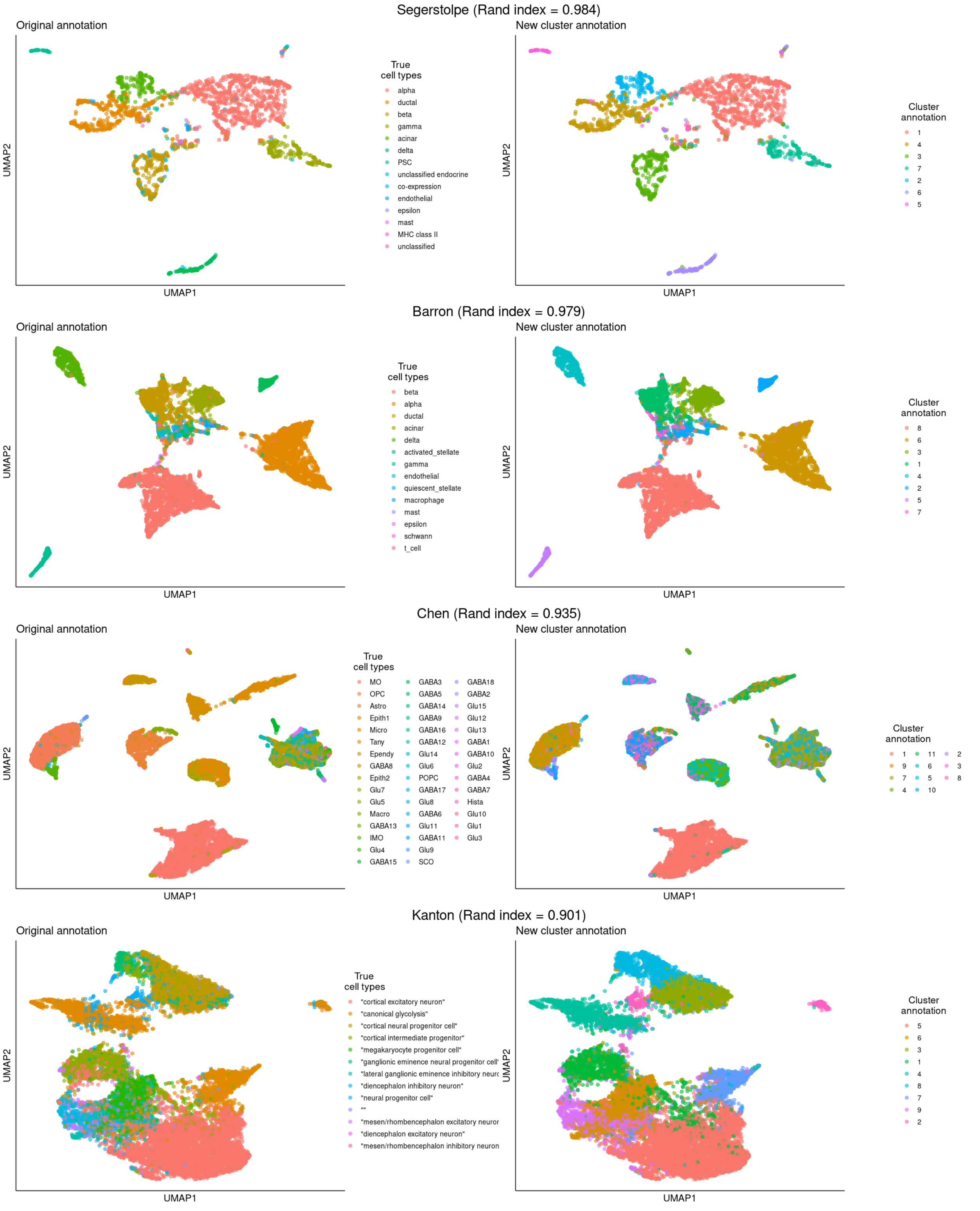

Figure S9. Visualization of the Segerstolpe, Baron, Chen, and Kanton raw datasets (top to bottom) using UMAP. For each dataset, the left panels shows UMAP plot with original labels and the righ panel shows UMAP plot with cluster annotations identified by scCAN. Different colors codes indicate different cell types and clusters.

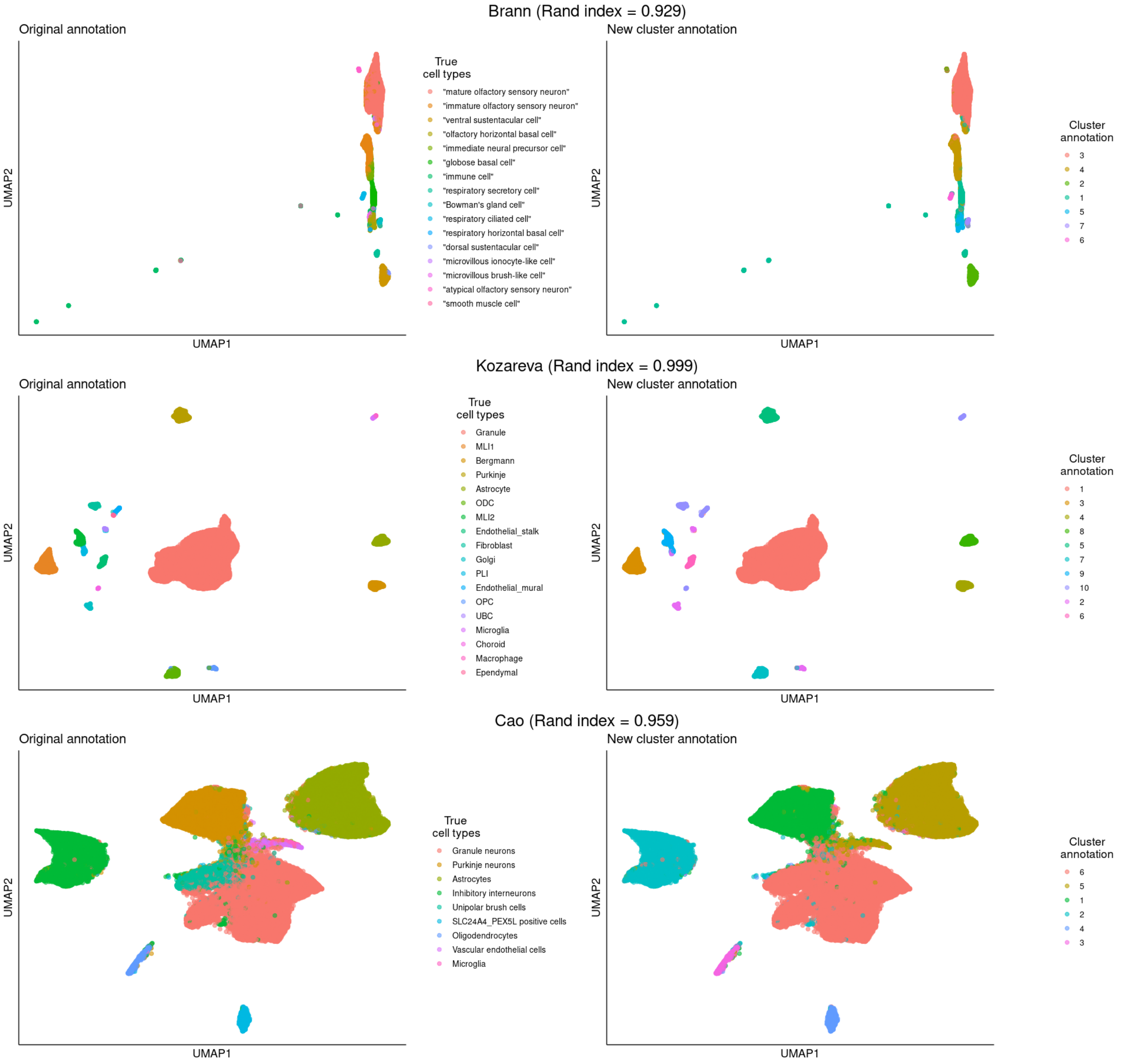

Figure S10. Visualization of the Brann, Kozareva, and Cao raw datasets (top to bottom) using UMAP. For each dataset, the left panels shows UMAP plot with original labels and the righ panel shows UMAP plot with cluster annotations identified by scCAN. Different colors codes indicate different cell types and clusters.

7 Comparison of the clustering methods used in Modules 2 and 3

The first method (core method) is more accurate but it requires more computational power and memory. Therefore, we developed the second method that allows users to analyze large datasets faster and using less memory. If the input dataset is small (by default 5,000 cells or less), both methods will be the same and thus produce the same results. When the dataset is large (5,000 cells or more), we use the first method to analyze a subset of the data to determine the cell types and then assign the the remaining cells to the determined cell types (second method).

Note that the default value of 5,000 allows us to have a sufficiently large sample size to properly determine the cell types which in turns will lead to a proper classification of the remaining cells. At the same time, 5,000 is a reasonable small number of samples that allows users to perform the analysis efficiently using personal computers. Users can also change this parameter to use the first method even for large datasets, if they have more memory and are willing to wait longer for their results. In the following text, as requested, we will provide a direct comparison between the two methods in terms of both accuracy and running time.

Table S8 shows a direct comparison of the two methods in terms of both accuracy and running time using the same server (with 200 GB of RAM). Consistent with the previous submission, we used adjusted Rand index (ARI), adjusted mutual information (AMI), and V-measure to assess the performance of each method. Cells with NA values indicate that a method was not able to analyze the dataset (out-of-memory). Cells highlighted in bold have the higher accuracy (ARI, AMI, and V-measure) and lower running time.

Overall, the first method can only analyze the first 21 datasets. It returns NA for the last seven datasets with 44,808 cells or more (out of memory). The second method can analyze all datasets, even for the Cao dataset with more than a million cells.

Regarding running time, the second method is substantially faster than the first method. For example, the second method was able to analyze the Zilionis dataset in 18 minutes while it takes the first method method almost 3 days. For the Cao dataset with a million cells, the second method finished the analysis in less than 40 minutes whereas the first method ran out of memory and could not analyze the data.

Regarding the accuracy, the first method is slightly more accurate (when they can analyze the data) but the difference between the two methods is marginal. For example, the first method has a higher ARI in three dataset (Guo, Chen, and Slyper) but has lower ARI in three other datasets (Montoro, Kanton, and Zilionis). Similarly, the two methods have comparable AMI and V-measure values.

In summary, the first method is slightly more accurate but the second method is capable of analyzing large datasets and requires less memory and running time. Therefore, the scCAN software automatically switches to the second method when analyzing datasets with 5,000 cells or more. Users can adjust this parameter if they wish to run the first method for larger datasets, given that they have sufficient memory and are willing to wait longer for the results.

Table S8. Performance of the two clustering methods used in Module 2 (method 1) and Module 3 (method 2) on single-cell datasets measured by adjusted Rand index (ARI), adjusted mutual information (AMI), V-measure and running time (minutes). Cells with NA values indicate that the method was not able to analyze the dataset (out-of-memory). Cells highlighted in bold have the higher accuracy (ARI, AMI, and V-measure) or lower running time.

**Datasets**	**#Cells**	**ARI**		**AMI**		**V-measure**		**Running Time**	
		**Method 1**	**Method 2**	**Method 1**	**Method 2**	**Method 1**	**Method 2**	**Method 1**	**Method 2**
**Pollen**	301	0.92	0.92	0.93	0.93	0.96	0.96	1.3	1.3
**Patel**	430	0.86	0.86	0.84	0.84	0.84	0.84	1.1	1.1
**Wang**	457	0.83	0.83	0.75	0.75	0.81	0.81	1.3	1.3
**Li**	561	0.94	0.94	0.95	0.95	0.96	0.96	1.7	1.7
**Usoskin**	622	0.93	0.93	0.88	0.88	0.93	0.93	1.4	1.4
**Camp**	777	0.61	0.61	0.72	0.72	0.82	0.82	1.6	1.6
**Xin**	1,600	0.98	0.98	0.91	0.91	0.92	0.92	2.4	2.4
**Muraro**	2,126	0.91	0.91	0.87	0.87	0.87	0.87	3.4	3.4
**Segerstolpe**	2,209	0.95	0.95	0.88	0.88	0.92	0.92	3.6	3.6
**Romanov**	2,881	0.63	0.63	0.61	0.61	0.62	0.62	5.5	5.5
**Zeisel**	3,005	0.86	0.86	0.81	0.81	0.82	0.82	5.9	5.9
**Lake**	3,042	0.58	0.58	0.74	0.74	0.75	0.75	6.1	6.1
**Montoro**	7,193	0.68	**0.70**	0.54	**0.58**	0.63	**0.65**	163.9	**17.9**
**Guo**	7,416	**0.88**	0.86	**0.88**	0.87	**0.90**	0.89	192.8	**17.9**
**Baron**	8,569	0.94	0.94	**0.88**	0.87	**0.90**	0.89	280.0	**17.9**
**Chen**	12,089	**0.85**	0.72	**0.69**	0.55	**0.77**	0.60	674.9	**17.9**
**Slyper**	13,316	**0.75**	0.67	**0.78**	0.73	**0.76**	0.73	777.7	**17.9**
**Kanton**	17,542	0.29	**0.67**	0.31	**0.64**	0.42	**0.64**	1,349	**17.9**
**Brann**	26,766	0.86	0.86	**0.73**	0.72	0.80	0.80	1,728	**17.9**
**Zilionis**	34,558	0.87	**0.89**	0.84	0.84	0.85	**0.89**	3,834	**18.5**
**Macosko**	44,808	NA	**0.89**	NA	**0.66**	NA	**0.70**	NA	**18.5**
**Hrvatin**	48,266	NA	**0.78**	NA	**0.76**	NA	**0.82**	NA	**18.6**
**Orozco**	100,055	NA	**0.77**	NA	**0.65**	NA	**0.75**	NA	**37.6**
**Miller**	142,523	NA	**0.90**	NA	**0.82**	NA	**0.88**	NA	**36.0**
**Darrah**	162,490	NA	**0.47**	NA	**0.53**	NA	**0.63**	NA	**37.9**
**Kozareva**	611,034	NA	**1.00**	NA	**0.94**	NA	**0.96**	NA	**45.0**
**Cao**	1,092,000	NA	**0.89**	NA	**0.84**	NA	**0.90**	NA	**39.0**

8 Effects of min-max scaling

The min-max scaling is not a scRNA-seq normalization method and it is not intended to do so. We leave the step of data processinga and normalization completely up to the users. This min-max scaling added to our method is used on top of the already normalized data provided by users. Such scaling is frequently used in deep learning models[33–36] with the common purpose of reducing standard deviation and suppressing the effect of outliers. Below, we will demonstrate that the min-max scaling step improves the clustering performance without altering the transcriptome landscape.

To demonstrate the usefulness of this min-max scaling on clustering, we re-analyzed all single-cell datasets using scCAN without applying the min-max scaling step. Figure S11 shows the ARI values obtained from scCAN in two scenarios: scCAN with and without the scaling step. Overall, the min-max scaling makes the analysis more robust (lower variance) and more accurate (higher ARI). This result demonstrates the usefulness of the min-max scaling in improving the performance of scCAN.

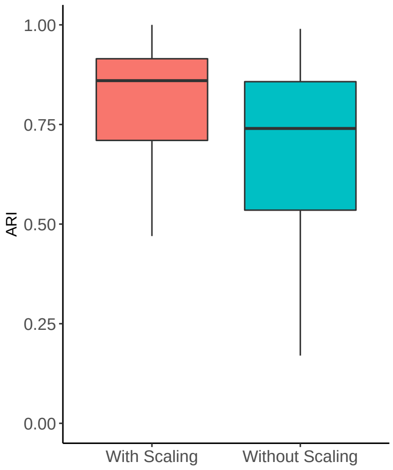

Figure S11. Impact of min-max scaling on scCAN. The analysis without scaling has higher variability and lower ARI values.

To further demonstrate that the min-max scaling does not alter the transcriptome landscape of the data, we calculated the distance correlation index ($dCor$)[37] between the two dimensional representation of scaling and non-scaling data generated by t-SNE. Given X and Y as the 2D representation of the scaling and non-scaling data, $dCor$ is calculated as $dCor = \frac{dCov(X,Y)}{\sqrt{dVar(X)dVar(Y)}}$ where $dCov(X,Y)$ is the distance covariance between X and Y while $dVar(X)$ and $dVar(Y)$ are distance variances of X and Y. Specifically, $dCor$ first calculates the pair-wise distances for X by computing the distance between each pair of cells, resulting in a square matrix. Second, it calculates the pair-wise distances for Y. Finally, it compares the two matrices using the formula described above to obtain the distance correlation. The $dCor$ coefficient has values ranging from 0 to 1, with the $dCor$ is expected to be 1 for a perfect similarity. In our analysis, when we rotate the transcriptome landscape, $dCor$ does not change. We re-analyzed the single-cell datasets and calculate the distance correlation for each dataset. Overall, the $dCor$ values obtained from all datasets are very high (median $dCor$ of 0.99 and variance of 0.01). This confirms that the min-max scaling does not alter the transcriptome landscape of the data while improving the clustering results.

9 Rare cell types detection

The sampling process is necessary to reduce both time and space complexity, but it can alter the capability of detecting rare cell types. By selecting 5,000 cells from a large dataset, we might end up with insufficient number of rare cells, and therefore reduce the chance of detecting them.

In addition, we have developed two strategies to enhance the method's capability of detecting rare cell types. First, we now allow users to change the parameter *samp.size* so that they can increase the sample size, thus boosting the method's capability in detecting rare cell types. Second, we provide an instruction to perform multi-state clustering, i.e., further splitting the clustering results. When a cell type has too few cells, these cells are often mistakenly grouped with other cell types. By further splitting each clusters, we are able to detect rare cell types that would not be possible by performing one-stage clustering.

To demonstrate the efficiency of both solutions, we have tested them on the Zilionis dataset. The Zilionis dataset has 34,558 cells and 9 cell types. The transcriptome landscape and the cell types of the dataset are shown in Figure S12A. Among the 9 cell types, the tRBC cell type has only 108 cells (0.3%). A sub-sample of 5,000 cells is expected to have approximately 19 tRBC cells, which might be insufficient for many clustering method to detect them. Indeed, as show in Figure S12B, scCAN mistakenly grouped tRBC cells with tPlasma cells when we used the default setting of $samp.size = 5,000$.

To demonstrate the efficiency of the first strategy, we set $samp.size = 10,000$. The clustering results using the new parameter is shown in Figure S12C. With a sample size of 10,000, the method can properly separate tRBC cells and assigned them to cluster 2. To quantify how well the method separates tRBC cells from other cells, we calculated the F1 score[38]. Briefly, $F1 = 2 * \frac{precison * recall}{precison + recall} = \frac{TP}{TP + \frac{1}{2}(FP + FN)}$ where: i) TP are tRBC cells that were correctly assigned to cluster 2, ii) FP are cells of other cell types that were mistakenly assigned to cluster 2, iii) and FN are tRBC cells but were not assigned to cluster 2. In the ideal case, FP=FN=0 which leads to F1=1. In the analysis shown in Figure S12C, F1 score is 0.9 which indicates that scCAN properly separated tRBC from the rest. The method is expected to perform even better if we further increase the sample size.

To demonstrate the efficiency of the second strategy, we performed a two-stage clustering using the the default setting of $samp.size = 5,000$. In stage one, we partitioned the data using scCAN and obtained the clustering results as shown in Figure S12B. In stage two, we further partitioned each cluster obtained from stage one using the same method scCAN. The results of stage two are shown in Figure S12D. Cluster 2 were further split into two sub-clusters: 2_1 and 2_2. The tRBC cells were completely separated from the rest (cluster 2_2) with an F1 score of 1. This demonstrates that users can efficiently detect rare cell types using multi-stage clustering even with the default parameter $samp.size = 5,000$.

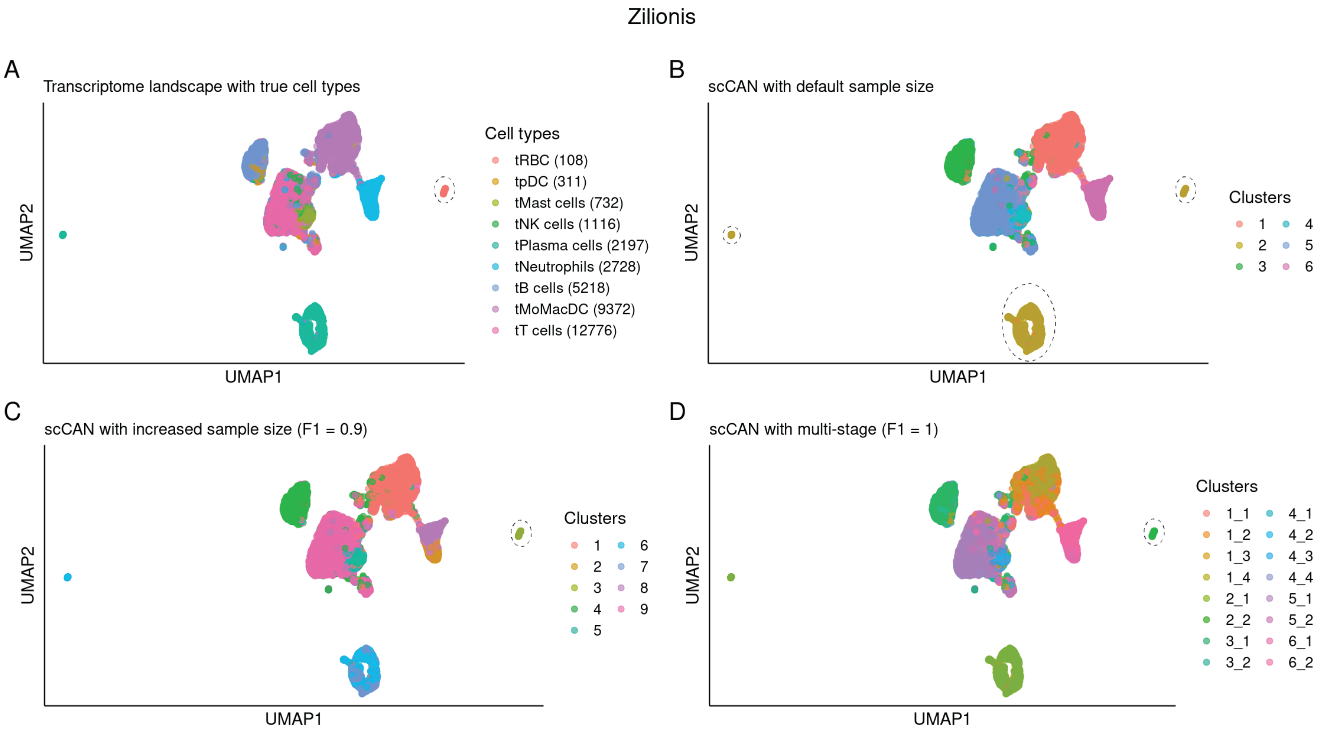

Figure S12. Rare cell type detection using the Zilionis dataset as example. The dataset has a total of 34,558 cells, in which there are 108 tRBC cells (rare cell type with 0.3% prevalence). (A) Transciptome landscape and true cell types. (B) Clustering results using scCAN with default sample size ($samp.size = 5,000$), in which tRBC are mistakenly grouped with tPlasma cells. (C) Clustering results with sample size of 10,000 ($samp.size = 10,000$). In this case, scCAN properly separates tRBC cells in cluster 2 with an F1 score of 0.9. (D) Clustering results using two-stage strategy and default sample size ($samp.size = 5,000$). scCAN properly separates tRBC cells in cluster 2 with a perfect F1 score of 1.

10 Scalability of scCAN

To demonstrate the scalability of scCAN, we downloaded and analyzed the Brain 1.3M dataset (`https://genomebiology.biomedcentral.com/articles/10.1186/s13059-017-1382-0`). Only scCAN and SCANPY were able to analyze this dataset of 1.3 million of cells. The clustering results of the two methods are shown in Figure S13. scCAN partitioned the data into 19 cluster whereas SCANPY partitioned the data into 20 clusters. The running time of scCAN and SCANPY were 51 minutes and 70 minutes, respectively. Note that we could not assess the accuracy of the two methods using this particular dataset because it does not have true cell type information.

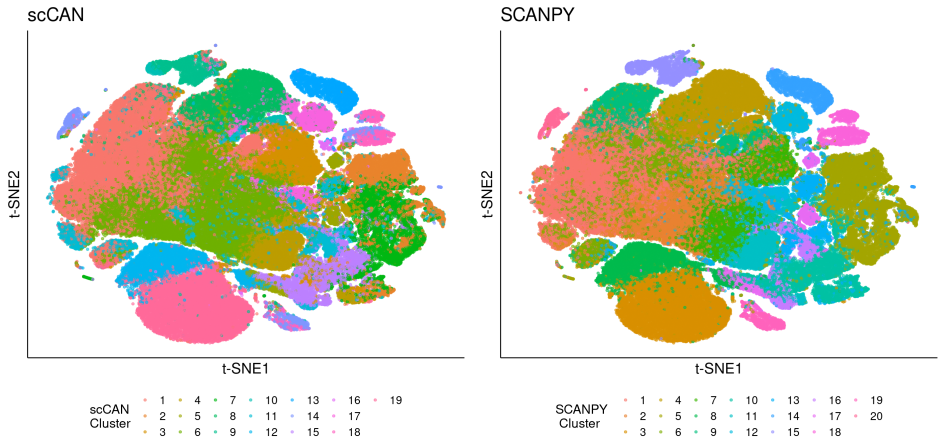

Figure S13. Clustering results of the Brain 1.3M dataset using scCAN and SCANPY. The left panel shows cell annotation of 20 clusters discovered by SCANPY. The right panel shows the cell partitions of 19 clusters identified from scCAN.

Second, we downloaded the Cao dataset[27] that contains 1,092,000 cells sequenced from the human cerebellum with known cell types. Again, only scCAN and SCANPY were able to analyze this dataset. Figure S14A shows the visualization of 2D t-SNE embedding data generated from raw data with original cells annotations while Figure S14B–C show the visualizations of Cao dataset using clusters generated from SCANPY and scCAN. SCANPY can cluster the whole dataset in 51 minutes with the ARI of 0.48 (Figure S14B), while scCAN takes 39 minutes to partition cells with the ARI of 0.89 (Figure S14C). We have updated the analysis results for the Brain 1.3M and Cao dataset to the main Manuscript and Supplementary Material.

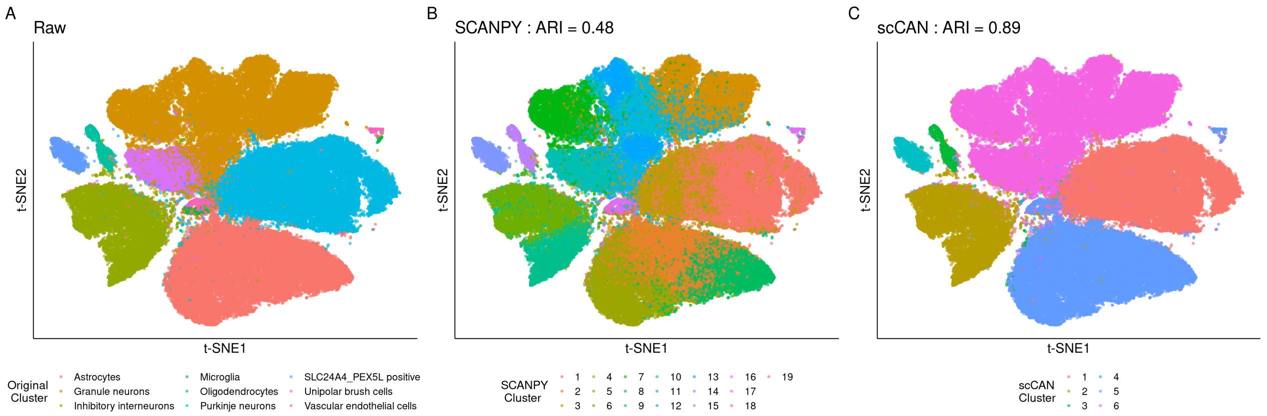

Figure S14. Visualizing of the Cao dataset using t-SNE. (A) Transcriptome landscape with true cell type information. (B) Transcriptome landscape of the clusters identified by SCANPY. (C) Transciptome landscape of clusters identified by scCAN. scCAN outperforms SCANPY by having a higher ARI value.

References

1. Barrett, T. *et al.* NCBI GEO: archive for functional genomics data sets–update. *Nucleic Acids Res.* **41**, D991–D995 (2013).
2. Pollen, A. A. *et al.* Low-coverage single-cell mRNA sequencing reveals cellular heterogeneity and activated signaling pathways in developing cerebral cortex. *Nat. Biotechnol.* **32**, 1053–1058, DOI: 10.1038/nbt.2967 (2014).
3. Patel, A. P. *et al.* Single-cell RNA-seq highlights intratumoral heterogeneity in primary glioblastoma. *Science* **344**, 1396–1401, DOI: 10.1126/science.1254257 (2014).
4. Wang, Y. J. *et al.* Single-cell transcriptomics of the human endocrine pancreas. *Diabetes* **65**, 3028–3038, DOI: 10.2337/db16-0405 (2016).
5. Li, H. *et al.* Reference component analysis of single-cell transcriptomes elucidates cellular heterogeneity in human colorectal tumors. *Nat. Genet.* **49**, 708 (2017).
6. Usoskin, D. *et al.* Unbiased classification of sensory neuron types by large-scale single-cell RNA sequencing. *Nat. Neurosci.* **18**, 145–153, DOI: 10.1038/nn.3881 (2015).
7. Camp, J. G. *et al.* Multilineage communication regulates human liver bud development from pluripotency. *Nature* **546**, 533–538, DOI: 10.1038/nature22796 (2017).
8. Xin, Y. *et al.* RNA sequencing of single human islet cells reveals type 2 diabetes genes. *Cell Metab.* **24**, 608–615, DOI: 10.1016/j.cmet.2016.08.018 (2016).
9. Muraro, M. J. *et al.* A single-cell transcriptome atlas of the human pancreas. *Cell Syst.* **3**, 385–394.e3, DOI: 10.1016/j.cels.2016.09.002 (2016).
10. Segerstolpe, Å. *et al.* Single-cell transcriptome profiling of human pancreatic islets in health and type 2 diabetes. *Cell Metab.* **24**, 593–607 (2016).
11. Romanov, R. A. *et al.* Molecular interrogation of hypothalamic organization reveals distinct dopamine neuronal subtypes. *Nat. Neurosci.* **20**, 176–188, DOI: 10.1038/nn.4462 (2017).
12. Zeisel, A. *et al.* Cell types in the mouse cortex and hippocampus revealed by single-cell RNA-seq. *Science* **347**, 1138–1142, DOI: 10.1126/science.aaa1934 (2015).
13. Lake, B. B. *et al.* Neuronal subtypes and diversity revealed by single-nucleus RNA sequencing of the human brain. *Science* **352**, 1586–1590 (2016).
14. Montoro, D. T. *et al.* A revised airway epithelial hierarchy includes CFTR-expressing ionocytes. *Nature* **560**, 319, DOI: 10.1038/s41586-018-0393-7 (2018).
15. Guo, J. *et al.* The dynamic transcriptional cell atlas of testis development during human puberty. *Cell Stem Cell* **26**, 262–276 (2020).
16. Baron, M. *et al.* A single-cell transcriptomic map of the human and mouse pancreas reveals inter-and intra-cell population structure. *Cell Syst.* **3**, 346–360 (2016).
17. Chen, R., Wu, X., Jiang, L. & Zhang, Y. Single-cell RNA-seq reveals hypothalamic cell diversity. *Cell Reports* **18**, 3227–3241, DOI: 10.1016/j.celrep.2017.03.004 (2017).
18. Kanton, S. *et al.* Organoid single-cell genomic atlas uncovers human-specific features of brain development. *Nature* **574**, 418–422 (2019).

19. Brann, D. H. *et al.* Non-neuronal expression of SARS-CoV-2 entry genes in the olfactory system suggests mechanisms underlying COVID-19-associated anosmia. *Science Advances* **6**, eabc5801 (2020).

20. Zilionis, R. *et al.* Single-cell transcriptomics of human and mouse lung cancers reveals conserved myeloid populations across individuals and species. *Immunity* **50**, 1317–1334 (2019).

21. Macosko, E. Z. *et al.* Highly parallel genome-wide expression profiling of individual cells using nanoliter droplets. *Cell* **161**, 1202–1214, DOI: 10.1016/j.cell.2015.05.002 (2015).

22. Hrvatin, S. *et al.* Single-cell analysis of experience-dependent transcriptomic states in the mouse visual cortex. *Nat. Neurosci.* **21**, 120–129 (2018).

23. Orozco, L. D. *et al.* Integration of eQTL and a single-cell atlas in the human eye identifies causal genes for age-related macular degeneration. *Cell Reports* **30**, 1246–1259 (2020).

24. Miller, A. J. *et al.* In vitro and in vivo development of the human airway at single-cell resolution. *Developmental Cell* **53**, 117–128 (2020).

25. Darrah, P. A. *et al.* Prevention of tuberculosis in macaques after intravenous BCG immunization. *Nature* **577**, 95–102 (2020).

26. Kozareva, V. *et al.* A transcriptomic atlas of the mouse cerebellum reveals regional specializations and novel cell types. *bioRxiv* (2020).

27. Cao, J. *et al.* A human cell atlas of fetal gene expression. *Science* **370**, eaba7721 (2020).

28. Zheng, G. X. Y. *et al.* Massively parallel digital transcriptional profiling of single cells. *Nat. Commun.* **8**, 14049 (2017).

29. Hubert, L. & Arabie, P. Comparing partitions. *J. Classif.* **2**, 193–218 (1985).

30. Vinh, N. X., Epps, J. & Bailey, J. Information theoretic measures for clusterings comparison: Variants, properties, normalization and correction for chance. *The J. Mach. Learn. Res.* **11**, 2837–2854 (2010).

31. Rosenberg, A. & Hirschberg, J. V-measure: A conditional entropy-based external cluster evaluation measure. In *Proceedings of the 2007 Joint Conference on Empirical Methods in Natural Language Processing and Computational Natural Language Learning (EMNLP-CoNLL)*, 410–420 (2007).

32. John, J. & Draper, N. R. An alternative family of transformations. *J. Royal Stat. Soc. Ser. C (Applied Stat.* **29**, 190–197 (1980).

33. He, K., Zhang, X., Ren, S. & Sun, J. Deep residual learning for image recognition. In *2016 IEEE Conference on Computer Vision and Pattern Recognition (CVPR)*, 770–778 (2016).

34. Huang, G., Liu, Z., Van Der Maaten, L. & Weinberger, K. Q. Densely connected convolutional networks. In *2017 IEEE Conference on Computer Vision and Pattern Recognition (CVPR)*, 4700–4708 (2017).

35. Szegedy, C. *et al.* Going deeper with convolutions. In *2015 IEEE Conference on Computer Vision and Pattern Recognition (CVPR)*, 1–9 (2015).

36. Tan, M. & Le, Q. EfficientNet: Rethinking model scaling for convolutional neural networks. In *Proceedings of the 36th International Conference on Machine Learning*, vol. 97, 6105–6114 (Long Beach, California, USA, 2019).

37. Székely, G. J., Rizzo, M. L. & Bakirov, N. K. Measuring and testing dependence by correlation of distances. *The Annals Stat.* **35**, 2769–2794 (2007).

38. Goutte, C. & Gaussier, E. A probabilistic interpretation of precision, recall and F-score, with implication for evaluation. In *European conference on information retrieval*, 345–359 (Springer, 2005).
